# Centralizers of Nilpotent Elements in Basic Classical Lie Superalgebras in Good Characteristic

**DOI:** 10.1007/s00031-023-09814-3

**Published:** 2023-09-12

**Authors:** Leyu Han

**Affiliations:** School of Mathematics, University of Birmingham, Birmingham, B15 2TT UK

**Keywords:** Basic classical Lie superalgebras, Nilpotent elements, Reachable elements, 17B05, 17B20, 17B22, 17B25

## Abstract

Let $\mathfrak {g}=\mathfrak {g}_{\bar{0}}\oplus \mathfrak {g}_{\bar{1}}$ be a basic classical Lie superalgebra over an algebraically closed field $\mathbb {K}$ whose characteristic $p>0$ is a good prime for $\mathfrak {g}$. Let $G_{\bar{0}}$ be the reductive algebraic group over $\mathbb {K}$ such that $\textrm{Lie}(G_{\bar{0}})=\mathfrak {g}_{\bar{0}}$. Suppose $e\in \mathfrak {g}_{\bar{0}}$ is nilpotent. Write $\mathfrak {g}^{e}$ for the centralizer of *e* in $\mathfrak {g}$ and $\mathfrak {z}(\mathfrak {g}^{e})$ for the centre of $\mathfrak {g}^{e}$. We calculate a basis for $\mathfrak {g}^{e}$ and $\mathfrak {z}(\mathfrak {g}^{e})$ by using associated cocharacters $\tau :\mathbb {K}^{\times }\rightarrow G_{\bar{0}}$ of *e*. In addition, we give the classification of *e* which are reachable, strongly reachable or satisfy the Panyushev property for exceptional Lie superalgebras $D(2,1;\alpha )$, *G*(3) and *F*(4).

## Introduction

Let $\mathfrak {g}=\mathfrak {g}_{\bar{0}}\oplus \mathfrak {g}_{\bar{1}}$ be a basic classical Lie superalgebra over an algebraically closed field $\mathbb {K}$ whose characteristic $p>0$ is a good prime for $\mathfrak {g}$. Note that the definition for a good prime is a natural extension of that for simple Lie algebras (see Definition 5). Let $e\in \mathfrak {g}_{\bar{0}}$ be nilpotent. We investigate the centralizer $\mathfrak {g}^{e}=\{x\in \mathfrak {g}:[e,x]=0\}$ of *e* in $\mathfrak {g}$ and the centre of centralizer $\mathfrak {z}(\mathfrak {g}^{e})=\{x\in \mathfrak {g}^{e}:[x,y]=0\text { for all }y\in \mathfrak {g}^{e}\}$ of *e* in $\mathfrak {g}$. A lot of research has been done on the centralizer and the centre of centralizer of nilpotent elements in the theory of Lie algebras. Although there are similarities between the theory of Lie superalgebras and the theory of Lie algebras, there is a lot less study in this direction in the case of Lie superalgebras and the structural theory of nilpotent orbits in Lie superalgebras remains to be better understood. In this paper, we calculate bases for $\mathfrak {g}^{e}$ and $\mathfrak {z}(\mathfrak {g}^{e})$ and study various properties relating *e* with $\mathfrak {g}^{e}$ and $\mathfrak {z}(\mathfrak {g}^{e})$.

**Table 1:** Algebraic groups $G_{\bar{0}}$

Lie superalgebras $\mathfrak {g}$	Algebraic groups $G_{\bar{0}}$
$\mathfrak {s}\mathfrak {l}(m|n),m\ne n$	$\left\{ (A,B)\in \textrm{GL}_{m}(\mathbb {K})\times \textrm{GL}_{n}(\mathbb {K}):\textrm{det}(A)=\textrm{det}(B)\right\}$
$\mathfrak {p}\mathfrak {s}\mathfrak {l}(n|n)$	$\left\{ (A,B)\in \textrm{GL}_{n}(\mathbb {K})\times \textrm{GL}_{n}(\mathbb {K}):\textrm{det}(A)=\textrm{det}(B)\}/\left\{ aI_{n|n}:a\in \mathbb {K}^{\times }\right\} \right\}$
$\mathfrak {o}\mathfrak {s}\mathfrak {p}(m|2n)$	$\textrm{O}_{m}(\mathbb {K})\times \textrm{Sp}_{2n}(\mathbb {K})$
$D(2,1;\alpha )$	$\textrm{SL}_{2}(\mathbb {K})\times \textrm{SL}_{2}(\mathbb {K})\times \textrm{SL}_{2}(\mathbb {K})$
*G*(3)	$\textrm{SL}_{2}(\mathbb {K})\times G_{2}$
*F*(4)	$\textrm{SL}_{2}(\mathbb {K})\times \textrm{Spin}_{7}(\mathbb {K})$

Research on the centralizer of nilpotent elements and their centres in the case of Lie algebras has been intensively developed since Springer [20] considered the centralizer $G^{u}$ of a unipotent element *u* in a simple algebraic group *G*. Many mathematicians undertook further study of $G^{u}$, the reader is referred to the introduction of [16] for an overview of research on $G^{u}$. For classical Lie algebras over an algebraically closed field of arbitrary characteristic, Jantzen gave an explicit account of the structure of $\mathfrak {g}^{e}$ in [12] and Yakimova worked out bases for $\mathfrak {z}(\mathfrak {g}^{e})$ in [22]. In [16], Lawther–Testerman dealt with the centralizer $G^{u}$ and its centre $Z(G^{u})$ over a field of characteristic 0 or a good prime based on Yakimova’s results. In [6, 7], the author identified $\mathfrak {g}^{e}$ and $\mathfrak {z}(\mathfrak {g}^{e})$ for basic classical Lie superalgebras over a field of characteristic zero and obtained analog of results of Lawther–Testerman [16] for those Lie superalgebras.

Define $\mathfrak {g}_{\mathbb {C}}$ to be a finite-dimensional basic classical Lie superalgebra over $\mathbb {C}$ and write $\Phi$ for a root system of $\mathfrak {g}_{\mathbb {C}}$. By [11, Theorem 3.9], there exists a Chevalley basis $\mathfrak {B}=\{e_{\alpha }:\alpha \in \Phi \}\cup \{h_{i}:1\le i\le s\}$ of $\mathfrak {g}_{\mathbb {C}}$ such that $[h_{i},e_{\alpha }]=\langle \alpha ,\alpha _{i}\rangle e_{\alpha }$ and $[e_{\alpha },e_{\beta }]=N_{\alpha ,\beta }e_{\alpha +\beta }$ where $\langle \alpha ,\alpha _{i}\rangle$ is defined in Eq. 2.4 and $N_{\alpha ,\beta }\in \mathbb {Z}$ can be determined explicitly. Let $\mathfrak {g}_{\mathbb {Z}}\subseteq \mathfrak {g}_{\mathbb {C}}$ be the Chevalley $\mathbb {Z}$-form of $\mathfrak {g}_{\mathbb {C}}$, i.e., $\mathfrak {g}_{\mathbb {Z}}$ is the $\mathbb {Z}$-span of $\mathfrak {B}$. Denote by $\mathfrak {h}_{\mathbb {Z}}=\langle h_{i}:1\le i\le s\rangle_{\mathbb {Z}}$ a Cartan subalgebra of $\mathfrak {g}_{\mathbb {Z}}$. We can view $\mathfrak {g}=\mathfrak {g}_{\mathbb {K}}$ where $\mathfrak {g}_{\mathbb {K}}=\mathbb {K}\otimes _{\mathbb {Z}}\mathfrak {g}_{\mathbb {Z}}$. It is natural to ask what is the structure of $\mathfrak {g}^{e}$ and $\mathfrak {z}(\mathfrak {g}^{e})$ in case $p>0$ is a good prime for $\mathfrak {g}$. For $\mathfrak {g}=\mathfrak {gl}(m|n)$, the construction of $\mathfrak {g}^{e}$ over a field of prime characteristic and that of zero characteristic are identical by [21] and [9]. However, the structure of $\mathfrak {g}^{e}$ in case of $\mathfrak {g}=\mathfrak {s}\mathfrak {l}(m|n)$ for $m\ne n$, $\mathfrak {p}\mathfrak {s}\mathfrak {l}(n|n)$, $\mathfrak {o}\mathfrak {s}\mathfrak {p}(m|2n)$ and three exceptional types have not been considered yet. In this paper, we aim to give a description of $\mathfrak {g}^{e}$ and further calculate the dimension of $\mathfrak {z}(\mathfrak {g}^{e})$ for the above types of Lie superalgebras.

In the remaining part of this introduction, we give a more detailed survey of our results.

Fix $\mathbb {K}$ an algebraically closed field with a good prime characteristic $p>0$, see Definition 5. Let $\mathfrak {g}=\mathfrak {g}_{\mathbb {K}}=\mathfrak {g}_{\bar{0}}\oplus \mathfrak {g}_{\bar{1}}$ be one of the Lie superalgebras in Table 2. Let $G_{\bar{0}}$ be the reductive algebraic group over $\mathbb {K}$ given as in Table 1 such that $\textrm{Lie}(G_{\bar{0}})=\mathfrak {g}_{\bar{0}}$. Then there is a representation $\rho :G_{\bar{0}}\rightarrow \textrm{GL}(\mathfrak {g}_{\bar{1}})$ such that $d_{\rho }:\textrm{Lie}(G_{\bar{0}})\rightarrow \mathfrak {g}\mathfrak {l}(\mathfrak {g}_{\bar{1}})$ determines the adjoint action of $\mathfrak {g}_{\bar{0}}$ on $\mathfrak {g}_{\bar{1}}$ (Table 2).

For each nilpotent element $e\in (\mathfrak {g}_{\mathbb {C}})_{\bar{0}}$, the Jacobson–Morozov Theorem allows one to associate an $\mathfrak {sl}_{2}$-triple $\{e,h,f\}\subseteq (\mathfrak {g}_{\mathbb {C}})_{\bar{0}}$ to *e*. According to [11, Section 3], the $\mathfrak {s}\mathfrak {l}_{2}$-triple can be chosen such that $\{e,h,f\}\subseteq (\mathfrak {g}_{\mathbb {Z}})_{\bar{0}}$ where $h\in \mathfrak {h}_{\mathbb {Z}}$ is of the form $h=\sum _{i=1}^{s}c_{i}h_{i}$ for $c_{i}\in \mathbb {Z}$ and $e=\sum _{\alpha \in \Phi }d_{i}e_{\alpha }$ for $d_{i}\in \mathbb {Z}$. Note that the $\textrm{ad}h$-grading of $\mathfrak {g}_{\mathbb {Z}}=\bigoplus _{j\in \mathbb {Z}}\mathfrak {g}_{\mathbb {Z}}(j;\textrm{ad}h)$ is given by $\mathfrak {g}_{\mathbb {Z}}(j;\textrm{ad}h)=\{x\in \mathfrak {g}_{\mathbb {Z}}:[h,x]=jx\}$ and this grading can be extended to $\mathfrak {g}_{\mathbb {C}}$. By the representation theory of $\mathfrak {s}\mathfrak {l}_{2}(\mathbb {C})$, we can determine $\textrm{ad}h$-eigenvalues of elements of $\mathfrak {g}_{\mathbb {C}}^{e}$. Based on the choice of *e*, we also can view $e\in \mathfrak {g}_{\mathbb {K}}$. We calculate bases for $\mathfrak {g}^{e}$ and $\mathfrak {z}(\mathfrak {g}^{e})$ for Lie superalgebras of type *A*(*m*, *n*), *B*(*m*, *n*), *C*(*n*), *D*(*m*, *n*) and $D(2,1;\alpha ), G(3), F(4)$ in Sects. 3–4 and 5 respectively. In particular, we have the following result (Table 3).

### Theorem 1

There exists a basis $\mathfrak {B}^{e}\subseteq \mathfrak {g}_{\mathbb {Z}}$ of $\mathfrak {g}_{\mathbb {C}}^{e}$ such that $\mathfrak {B}^{e}$ viewed in $\mathfrak {g}^{e}$ is a basis for $\mathfrak {g}^{e}$. Similarly, we can find a basis $\mathfrak {B}_{\mathfrak {z}}^{e}\subseteq \mathfrak {g}_{\mathbb {Z}}$ of $\mathfrak {z}(\mathfrak {g}_{\mathbb {C}}^{e})$ such that $\mathfrak {B}_{\mathfrak {z}}^{e}$ viewed in $\mathfrak {z}(\mathfrak {g}^{e})$ is a basis for $\mathfrak {z}(\mathfrak {g}^{e})$. Note that when $\mathfrak {g}=\mathfrak {s}\mathfrak {l}(m|n)$ for $m\ne n$ or $\mathfrak {p}\mathfrak {s}\mathfrak {l}(n|n)$ for $n>1$, we require that $\textrm{char}(\mathbb {K})=p$ does not divide *m* and *n*.

Note that in good characteristic there is a substitute for $\mathfrak {s}\mathfrak {l}_{2}$-triples, so called associated cocharacters, see Definition 6 below. Let $\tau :\mathbb {K}^{\times }\rightarrow G_{\bar{0}}$ be a cocharacter associated to *e*. Denote by $\mathfrak {g}=\bigoplus _{j\in \mathbb {Z}}\mathfrak {g}(j;\tau )$ the $\tau$-grading on $\mathfrak {g}$ where $\mathfrak {g}(j;\tau )=\{x\in \mathfrak {g}:\textrm{Ad}(\tau (t))(x)=t^{j}x\text { for all }t\in \mathbb {K}^{\times }\}$. In [21, Section 3], Wang–Zhao studied properties of the $\tau$-grading on $\mathfrak {g}$ with some restrictions on *p*. Combining Theorem 1 with Lemma 8, we obtain the following theorem which gives a more general statement on the $\tau$-grading on $\mathfrak {g}$ (Table 4).

### Theorem 2

Let $\mathfrak {g}=\mathfrak {g}_{\bar{0}}\oplus \mathfrak {g}_{\bar{1}}$ be one of the basic classical Lie superalgebras in Table 2 and $e\in \mathfrak {g}_{\bar{0}}$ be nilpotent. Then the cocharacter $\tau :\mathbb {K}^{\times }\rightarrow G_{\bar{0}}$ associated to *e* defines a $\mathbb {Z}$-grading $\mathfrak {g}=\bigoplus _{j\in \mathbb {Z}}\mathfrak {g}(j;\tau )$ such that $\mathfrak {g}^{e}=\bigoplus _{j\ge 0}\mathfrak {g}^{e}(j;\tau )$ and $\dim \mathfrak {g}^{e}(j;\tau )=\dim \mathfrak {g}(j;\tau )-\dim \mathfrak {g}((j+2);\tau )$ for $j\ge 0$.

Our next result focuses on the $\mathbb {Z}$-grading on $\mathfrak {z}(\mathfrak {g}^{e})$. In the case of simple Lie algebras, the nilpotent element *e* spans the degree 2 part of the $\textrm{ad}h$-grading of the centre of centralizer of *e*. It is known as the Brylinsky–Kostant theorem, see [1, 15]. Write $\mathfrak {z}(j;\textrm{ad}h)=\mathfrak {z}(\mathfrak {g}_{\mathbb {C}}^{e})\cap \mathfrak {g}(j;\textrm{ad}h)$ and $\mathfrak {z}(j;\tau )=\mathfrak {z}(\mathfrak {g}^{e})\cap \mathfrak {g}(j;\tau )$. Theorem 3 can be viewed as the Lie superalgebra version of the Brylinsky–Kostant theorem (Table 5).

### Theorem 3

Let $\mathfrak {g}=\mathfrak {g}_{\bar{0}}\oplus \mathfrak {g}_{\bar{1}}$ be one of the basic classical Lie superalgebras in Table 2 and $e\in \mathfrak {g}_{\bar{0}}$ be nilpotent. Then $\mathfrak {z}(2;\textrm{ad}h)=\langle e\rangle$ and $\mathfrak {z}(2;\tau )=\langle e\rangle$.

The element *e* is called *reachable* if $e\in [\mathfrak {g}^{e},\mathfrak {g}^{e}]$, such element were first considered by Elashvili and Grélaud and called *compact* in [3]. The element *e* is called *strongly reachable* if $\mathfrak {g}^{e}=[\mathfrak {g}^{e},\mathfrak {g}^{e}]$. We say that *e* satisfies the *Panyushev property* [18] if in the $\tau$-grading $\mathfrak {g}^{e}=\bigoplus _{j\ge 0}\mathfrak {g}(j)$, the subalgebra $\mathfrak {g}^{e}(\ge 1)=\bigoplus _{j\ge 1}\mathfrak {g}(j)$ is generated by $\mathfrak {g}^{e}(1)$. In the case of Lie algebras, Panyushev [18] showed that for $\mathfrak {g}$ of type $A_{n}$, a nilpotent element *e* is reachable if and only if *e* satisfies the Panyushev property. The result was extended to cases for $\mathfrak {g}$ of type $B_{n}$, $C_{n}$, $D_{n}$ by Yakimova [23] and for $\mathfrak {g}$ of exceptional types by de Graaf [2]. The author [8] gave the classification of even elements that are reachable, strongly reachable or satisfying the Panyushev property in basic classical Lie superalgebras over $\mathbb {C}$. Our final result extends the results in [8] to good characteristic, which illustrates the relation between the property of being reachable and the Panyushev property.

### Theorem 4

Let $\mathfrak {g}=\mathfrak {g}_{\bar{0}}\oplus \mathfrak {g}_{\bar{1}}$ be one of exceptional Lie superalgebras $D(2,1;\alpha )$, *G*(3), *F*(4) and $e\in \mathfrak {g}_{\bar{0}}$ be nilpotent. Then *e* is reachable if and only if *e* satisfies the Panyushev property except for $\mathfrak {g}=G(3)$ and $e=x_{1}$ or $e=E+x_{1}$. The nilpotent orbits that are reachable, strongly reachable or satisfying the Panyushev property are listed in Tables 14–16.

This paper is organized as follows. We first recall some fundamental concepts of Lie superalgebras such as basic classical Lie superalgebras, root systems and cocharacters associated to nilpotent elements in Sect. 2. For each system of positive roots, we identify the highest root in it. In Sects. 3–4, we determine bases of $\mathfrak {g}^{e}$ and $\mathfrak {z}(\mathfrak {g}^{e})$ for $\mathfrak {g}=\mathfrak {s}\mathfrak {l}(m|n)$ for $m\ne n$, $\mathfrak {p}\mathfrak {s}\mathfrak {l}(n|n)$ and $\mathfrak {o}\mathfrak {s}\mathfrak {p}(m|2n)$. Bases of $\mathfrak {g}^{e}$ and $\mathfrak {z}(\mathfrak {g}^{e})$ for an exceptional Lie superalgebra $\mathfrak {g}$ are given in Sect. 5. Using the structure of $\mathfrak {g}^{e}$, for an exceptional Lie superalgebra $\mathfrak {g}$ in Sect. 5, we determine which even nilpotent elements are reachable, strongly reachable or satisfy the Panyushev property in Sect. 6.

**Table 2:** Basic classical Lie superalgebras

$\mathfrak {g}=\mathfrak {g}_{\bar{0}}\oplus \mathfrak {g}_{\bar{1}}$	$\mathfrak {g}_{\bar{0}}$
$A(m,n)=\mathfrak {s}\mathfrak {l}(m|n)$, $m,n\ge 1$, $m\ne n$	$\mathfrak {s}\mathfrak {l}_{m}(\mathbb {K})\oplus \mathfrak {s}\mathfrak {l}_{n}(\mathbb {K})\oplus \mathbb {K}$
$A(n,n)=\mathfrak {p}\mathfrak {s}\mathfrak {l}(n|n)$, $n>1$	$\mathfrak {s}\mathfrak {l}_{n}(\mathbb {K})\oplus \mathfrak {s}\mathfrak {l}_{n}(\mathbb {K})$
$B(m,n)=\mathfrak {o}\mathfrak {s}\mathfrak {p}(m|2n)$, *m* is odd, $m,n\ge 1$	$\mathfrak {o}_{m}(\mathbb {K})\oplus \mathfrak {s}\mathfrak {p}_{2n}(\mathbb {K})$
$C(n)=\mathfrak {o}\mathfrak {s}\mathfrak {p}(2|2n)$, $n\ge 1$	$\mathfrak {o}_{2}(\mathbb {K})\oplus \mathfrak {s}\mathfrak {p}_{2n}(\mathbb {K})$
$D(m,n)=\mathfrak {o}\mathfrak {s}\mathfrak {p}(m|2n)$, $m\ge 4$ is even, $n\ge 1$	$\mathfrak {o}_{m}(\mathbb {K})\oplus \mathfrak {s}\mathfrak {p}_{2n}(\mathbb {K})$
$D(2,1;\alpha )$, $\alpha \in \mathbb {K}\backslash \{0,-1\}$	$\mathfrak {s}\mathfrak {l}_{2}(\mathbb {K})\oplus \mathfrak {s}\mathfrak {l}_{2}(\mathbb {K})\oplus \mathfrak {s}\mathfrak {l}_{2}(\mathbb {K})$
*G*(3)	$\mathfrak {s}\mathfrak {l}_{2}(\mathbb {K})\oplus G_{2}$
*F*(4)	$\mathfrak {s}\mathfrak {l}_{2}(\mathbb {K})\oplus \mathfrak {s}\mathfrak {o}_{7}(\mathbb {K})$

## Preliminaries and Notations

### Basic Classical Lie Superalgebras

Finite-dimensional simple Lie superalgebras over $\mathbb {C}$ were classified by Kac in [14]. Among those simple Lie superalgebras, we focus on basic classical Lie superalgebras in this paper. Recall that a finite-dimensional simple Lie superalgebra $\mathfrak {g}=\mathfrak {g}_{\bar{0}}\oplus \mathfrak {g}_{\bar{1}}$ is called *a basic classical Lie superalgebra* if the even part $\mathfrak {g}_{\bar{0}}$ is a reductive Lie algebra and there exists a non-degenerate supersymmetric invariant even bilinear form $(\cdot ,\cdot )$ on $\mathfrak {g}$. Note that these Lie superalgebras are well defined over $\mathbb {K}$ and remain to simple by [21, Section 2]. Below in Table 2, we recall the list of basic classical Lie superalgebras over $\mathbb {K}$ that are not Lie algebras, they are *A*(*m*, *n*) for $m\ne n$, *A*(*n*, *n*) for $n>1$, *B*(*m*, *n*), *C*(*n*), *D*(*m*, *n*) and three exceptional types $D(2,1;\alpha )$, *G*(3), *F*(4). The explicit construction of *A*(*m*, *n*), *A*(*n*, *n*), *B*(*m*, *n*), *C*(*n*), *D*(*m*, *n*) can be found for example in [6, Sections 3–4] and that of $D(2,1;\alpha )$, *G*(3), *F*(4) can be found for example in [7, Sections 4–6].

### Systems of Positive Roots and the Highest Root

Let $\mathfrak {g}$ be one of the basic classical Lie superalgebras in Table 2 and let $\Phi =\Phi _{\bar{0}}\cup \Phi _{\bar{1}}$ be a root system for $\mathfrak {g}$. Let $\Phi ^{+}$ be a system of positive roots and $\Pi =\{\alpha _{1},\dots ,\alpha _{l}\}$ be the corresponding system of simple roots. Note that the conjugacy classes of simple systems for basic classical Lie superalgebras were classified in [13]. Together with results in [6, 7], there is in general more than one system of simple roots. For each system of positive roots, there is an unique *highest root* $\widetilde{\alpha }$ in $\Phi ^{+}$ such that $\widetilde{\alpha }=\sum _{i=1}^{l}a_{i}\alpha _{i}$, and for any other $\beta =\sum _{i=1}^{l}b_{i}\alpha _{i}\in \Phi ^{+}$, we have $b_{i}\le a_{i}$ for all *i*.

In the following part of this subsection, we adopt notations in [6, Sections 3–4] and [7, Sections 4–6] for root systems of $\mathfrak {g}$ and systems of simple roots. In particular, we use the results in [6, 7] to determine the highest root in $\Phi ^{+}$ for each system of simple roots.

- **1.** *A*(*m*, *n*) Let $V=V_{\bar{0}}\oplus V_{\bar{1}}$ be a finite-dimensional vector space over $\mathbb {K}$ such that $\dim V_{\bar{0}}=m$ and $\dim V_{\bar{1}}=n$. For a homogeneous basis $\{v_{1},\dots ,v_{m+n}\}$ of *V*, we denote by $\eta _{i}$ the parity of $v_{i}$, i.e. $\eta _{i}=\bar{0}$ if $v_{i}\in V_{\bar{0}}$ and $\eta _{i}=\bar{1}$ if $v_{i}\in V_{\bar{1}}$. Let $e_{ij}$ be the *ij*-matrix unit. A Cartan subalgebra $\mathfrak {h}$ of $\mathfrak {g}\mathfrak {l}(m|n)$ has a basis $\{h_{i}=e_{i,i}:1\le i\le m+n\}$ and a dual basis $\{\varepsilon _{i}^{\eta _{i}}\in \mathfrak {h}^{*}:1\le i\le m+n\}$ is defined by $\varepsilon _{i}^{\eta _{i}}(h_{j})=\delta _{ij}$ such that $(\varepsilon _{i}^{\eta _{i}},\varepsilon _{j}^{\eta _{j}})=(-1)^{\eta _{i}}\delta _{ij}$. Then $\mathfrak {g}=\mathfrak {s}\mathfrak {l}(V)$ or $\mathfrak {p}\mathfrak {s}\mathfrak {l}(V)$ has a root system $\Phi =\Phi _{\bar{0}}\cup \Phi _{\bar{1}}$ such that $\Phi _{\bar{0}}=\{\varepsilon _{i}^{\eta _{i}}-\varepsilon _{j}^{\eta _{j}}:1\le i,j\le m+n,i\ne j,\eta _{i}=\eta _{j}\}$ and $\Phi _{\bar{1}}=\{\varepsilon _{i}^{\eta _{i}}-\varepsilon _{j}^{\eta _{j}}:1\le i,j\le m+n,i\ne j,\eta _{i}\ne \eta _{j}\}$. The system of simple roots is given by $\Pi =\{\alpha _{i}=\varepsilon _{i}^{\eta _{i}}-\varepsilon _{i+1}^{\eta _{i+1}}:1\le i<m+n\}$ and the highest root is $\widetilde{\alpha }=\varepsilon _{1}^{\eta _{1}}-\varepsilon _{m+n}^{\eta _{m+n}}=\sum _{i=1}^{m+n-1}\alpha _{i}$.
- **2.** *B*(*m*, *n*), *C*(*n*) **or** *D*(*m*, *n*) Let $V=V_{\bar{0}}\oplus V_{\bar{1}}$ be a finite-dimensional vector space over $\mathbb {K}$ such that $\dim V_{\bar{0}}=m$ and $\dim V_{\bar{1}}=2n$ and let $\mathfrak {g}=\mathfrak {o}\mathfrak {s}\mathfrak {p}(V)$. Write $l=\left\lfloor \frac{m}{2}\right\rfloor$. A Cartan subalgebra $\mathfrak {h}$ of $\mathfrak {g}$ has a basis $\{h_{i}=e_{i,i}-e_{-i,-i}:1\le i\le l+n\}$ and a dual basis $\{\varepsilon _{i}^{\eta _{i}}\in \mathfrak {h}^{*}:1\le i\le l+n\}$ is defined by $\varepsilon _{i}^{\eta _{i}}(h_{j})=\delta _{ij}$ such that $(\varepsilon _{i}^{\eta _{i}},\varepsilon _{j}^{\eta _{j}})=(-1)^{\eta _{i}}\delta _{ij}$. When *m* is odd, a root system for $\mathfrak {g}$ is $\Phi _{\bar{0}}=\{\pm \varepsilon _{i}^{\eta _{i}}\pm \varepsilon _{j}^{\eta _{j}}:i\ne j,\eta _{i}=\eta _{j}\}\cup \{\pm \varepsilon _{i}^{\bar{0}}\}\cup \{\pm 2\varepsilon _{i}^{\bar{1}}\}$ and $\Phi _{\bar{1}}=\{\pm \varepsilon _{i}^{\bar{1}}\}\cup \{\pm \varepsilon _{i}^{\eta _{i}}\pm \varepsilon _{j}^{\eta _{j}}:i\ne j,\eta _{i}\ne \eta _{j}\}$. A system of simple roots is $\{\alpha _{i}=\varepsilon _{i}^{\eta _{i}}-\varepsilon _{i+1}^{\eta _{i+1}}:1\le i\le l+n-1\}\cup \{\alpha _{l+n}=\varepsilon _{l+n}^{\eta _{l+n}}\}$. The associated system of positive roots is $\Phi ^{+}=\{\varepsilon _{i}^{\eta _{i}}\pm \varepsilon _{j}^{\eta _{j}},\varepsilon _{k}^{\eta _{k}},2\varepsilon _{t}^{\bar{1}}\}$, where the highest root is
  $$\begin{aligned} \widetilde{\alpha }=\left\{ \begin{array}{ll} \varepsilon _{1}^{\eta _{1}}+\varepsilon _{2}^{\eta _{2}}=\alpha _{1}+2\sum _{i=2}^{l+n}\alpha _{i} &{} \text {if }\eta _{1}=\bar{0};\\ 2\varepsilon _{1}^{\bar{1}}=2\sum _{i=1}^{l+n}\alpha _{i} &{} \text {if }\eta _{1}=\bar{1}. \end{array}\right.  \end{aligned}$$
  When *m* is even, a root system for $\mathfrak {g}$ is $\Phi _{\bar{0}}=\{\pm \varepsilon _{i}^{\eta _{i}}\pm \varepsilon _{j}^{\eta _{j}}:i\ne j,\eta _{i}=\eta _{j}\}\cup \{\pm 2\varepsilon _{i}^{\bar{1}}\}$ and $\Phi _{\bar{1}}=\{\pm \varepsilon _{i}^{\eta _{i}}\pm \varepsilon _{j}^{\eta _{j}}:i\ne j,\eta _{i}\ne \eta _{j}\}$. Note that there are two possibilities for the systems of simple roots with the same associated system of positive roots $\Phi ^{+}=\{\varepsilon _{i}^{\eta _{i}}\pm \varepsilon _{j}^{\eta _{j}},2\varepsilon _{t}^{\bar{1}}\}$. There are
  - $\Pi _{1}=\{\alpha _{i}=\varepsilon _{i}^{\eta _{i}}-\varepsilon _{i+1}^{\eta _{i+1}}:1\le i\le l+n-2\}\cup \{\alpha _{l+n-1}=\varepsilon _{l+n-1}^{\eta _{l+n-1}}-\varepsilon _{l+n}^{\bar{1}}\}\cup \{\alpha _{l+n}=2\varepsilon _{l+n}^{\bar{1}}\}$, and the highest root is
    $$\begin{aligned} \widetilde{\alpha }_{(1)}=\left\{ \begin{array}{ll} \varepsilon _{1}^{\eta _{1}}+\varepsilon _{2}^{\eta _{2}}=\alpha _{1}+2\sum _{i=2}^{l+n-1}\alpha _{i}+\alpha _{l+n} &{} \text {if }\eta _{1}=\bar{0};\\ 2\varepsilon _{1}^{\bar{1}}=2\sum _{i=1}^{l+n-1}\alpha _{i}+\alpha _{l+n} &{} \text {if }\eta _{1}=\bar{1}. \end{array}\right.  \end{aligned}$$
  - $\Pi _{2}=\{\alpha _{i}=\varepsilon _{i}^{\eta _{i}}-\varepsilon _{i+1}^{\eta _{i+1}}:1\le i\le l+n-2\}\cup \{\alpha _{l+n-1}=\varepsilon _{l+n-1}^{\eta _{l+n-1}}-\varepsilon _{l+n}^{\eta _{l+n}}\}\cup \{\alpha _{l+n}=\varepsilon _{l+n-1}^{\eta _{l+n-1}}+\varepsilon _{l+n}^{\eta _{l+n}}\}$, and the highest root is
    $$\begin{aligned} \widetilde{\alpha }_{(2)}=\left\{ \begin{array}{ll} \varepsilon _{1}^{\eta _{1}}+\varepsilon _{2}^{\eta _{2}}=\alpha _{1}+2\sum _{i=2}^{l+n-2}\alpha _{i}+\alpha _{l+n-1}+\alpha _{l+n} &{} \text {if }\eta _{1}=\bar{0};\\ 2\varepsilon _{1}^{\bar{1}}=2\sum _{i=1}^{l+n-2}\alpha _{i}+\alpha _{l+n-1}+\alpha _{l+n} &{} \text {if }\eta _{1}=\bar{1}. \end{array}\right.  \end{aligned}$$
- **3.** $D(2,1;\alpha )$ (**with** $\alpha \ne 0,-1$) The Lie superalgebra $\mathfrak {g}=D(2,1;\alpha )$ has a root system $\Phi =\Phi _{\bar{0}}\cup \Phi _{\bar{1}}$ such that $\Phi _{\bar{0}}=\lbrace \pm 2\beta _{1},\pm 2\beta _{2},\pm 2\beta _{3}\rbrace$, $\Phi _{\bar{1}}=\lbrace \pm \beta _{1}\pm \beta _{2}\pm \beta _{3}\rbrace$ where $\{\beta _{1},\beta _{2},\beta _{3}\}$ is an orthogonal basis of $\mathbb {R}\Phi$ such that $(\beta _{1},\beta _{1})=\frac{1}{2}$, $(\beta _{2},\beta _{2})=-\frac{1}{2}\alpha -\frac{1}{2}$ and $(\beta _{3},\beta _{3})=\frac{1}{2}\alpha$. According to [7, Subsection 4.2], there are four conjugacy classes of systems of simple roots which we list in the Table 3.

**Table 3:** The highest root in system of positive roots for $D(2,1;\alpha )$

System of simple roots $\Pi =\{\alpha _{1},\alpha _{2},\alpha _{3}\}$	Associated system of positive roots $\Phi ^{+}$	The highest root $\widetilde{\alpha }$
$\{2\beta _{1},-\beta _{1}+\beta _{2}-\beta _{3},2\beta _{3}\rbrace$	$\lbrace 2\beta _{i},\pm \beta _{1}+\beta _{2}\pm \beta _{3}:i=1,2,3\rbrace$	$2\beta _{2}=\alpha _{1}+2\alpha _{2}+\alpha _{3}$
$\{2\beta _{1},-\beta _{1}-\beta _{2}+\beta _{3},2\beta _{2}\rbrace$	$\lbrace 2\beta _{i},\pm \beta _{1}\pm \beta _{2}+\beta _{3}:i=1,2,3\rbrace$	$2\beta _{3}=\alpha _{1}+2\alpha _{2}+\alpha _{3}$
$\{2\beta _{3},\beta _{1}-\beta _{2}-\beta _{3},2\beta _{2}\rbrace$	$\lbrace 2\beta _{i},\beta _{1}\pm \beta _{2}\pm \beta _{3}:i=1,2,3\rbrace$	$2\beta _{1}=\alpha _{1}+2\alpha _{2}+\alpha _{3}$
$\{-\beta _{1}+\beta _{2}+\beta _{3},\beta _{1}-\beta _{2}+\beta _{3},\beta _{1}+\beta _{2}-\beta _{3}\rbrace$	$\lbrace 2\beta _{i},\pm \beta _{1}+\beta _{2}+\beta _{3},\beta _{1}-\beta _{2}+\beta _{3},\beta _{1}+\beta _{2}-\beta _{3}:i=1,2,3\rbrace$	$\beta _{1}+\beta _{2}+\beta _{3}=\alpha _{1}+\alpha _{2}+\alpha _{3}$

**Table 4:** The highest root in system of positive roots for *G*(3)

System of simple roots $\Pi =\{\alpha _{1},\alpha _{2},\alpha _{3}\}$	Associated system of positive roots $\Phi ^{+}$	The highest root $\widetilde{\alpha }$
$\{\delta +\varepsilon _{3},\varepsilon _{1},\varepsilon _{2}-\varepsilon _{1}\rbrace$	$\lbrace \varepsilon _{1},\varepsilon _{2},-\varepsilon _{3},\varepsilon _{2}-\varepsilon _{1},\varepsilon _{1}-\varepsilon _{3},\varepsilon _{2}-\varepsilon _{3},\delta ,2\delta ,\delta \pm \varepsilon _{i}:i=1,2,3\rbrace$	$2\delta =2\alpha _{1}+4\alpha _{2}+2\alpha _{3}$
$\{-\delta -\varepsilon _{3},\delta -\varepsilon _{2},\varepsilon _{2}-\varepsilon _{1}\rbrace$	$\lbrace \varepsilon _{1},\varepsilon _{2},-\varepsilon _{3},\varepsilon _{2}-\varepsilon _{1},\varepsilon _{1}-\varepsilon _{3},\varepsilon _{2}-\varepsilon _{3},\delta ,2\delta ,\delta \pm \varepsilon _{1},\delta \pm \varepsilon _{2},\pm \delta -\varepsilon _{3}\rbrace$	$\delta -\varepsilon _{3}=3\alpha _{1}+4\alpha _{2}+2\alpha _{3}$
$\{\delta ,-\delta +\varepsilon _{1},\varepsilon _{2}-\varepsilon _{1}\rbrace$	$\lbrace \varepsilon _{1},\varepsilon _{2},-\varepsilon _{3},\varepsilon _{2}-\varepsilon _{1},\varepsilon _{1}-\varepsilon _{3},\varepsilon _{2}-\varepsilon _{3},\delta ,2\delta ,\pm \delta +\varepsilon _{1},\pm \delta +\varepsilon _{2},\pm \delta -\varepsilon _{3}\rbrace$	$\varepsilon _{2}-\varepsilon _{3}=3\alpha _{1}+2\alpha _{2}+2\alpha _{3}$
$\{\varepsilon _{1},-\delta +\varepsilon _{2},\delta -\varepsilon _{1}\rbrace$	$\lbrace \varepsilon _{1},\varepsilon _{2},-\varepsilon _{3},\varepsilon _{2}-\varepsilon _{1},\varepsilon _{1}-\varepsilon _{3},\varepsilon _{2}-\varepsilon _{3},\delta ,2\delta ,\delta \pm \varepsilon _{1},\pm \delta +\varepsilon _{2},\pm \delta -\varepsilon _{3}\rbrace$	$\varepsilon _{2}-\varepsilon _{3}=3\alpha _{1}+2\alpha _{2}+2\alpha _{3}$

**Table 5:** The highest root in system of positiveroots for *F*(4)

System of simple roots $\Pi =\{\alpha _{1},\alpha _{2},\alpha _{3},\alpha _{4}\}$	Associated system of positive roots $\Phi ^{+}$	The highest root $\widetilde{\alpha }$
$\{\frac{1}{2}(\delta -\varepsilon _{1}-\varepsilon _{2}-\varepsilon _{3}),\varepsilon _{3},\varepsilon _{2}-\varepsilon _{3},\varepsilon _{1}-\varepsilon _{2}\}$	$\{\varepsilon _{1},\varepsilon _{2},\varepsilon _{3},\varepsilon _{1}\pm \varepsilon _{2},\varepsilon _{1}\pm \varepsilon _{3},\varepsilon _{2}\pm \varepsilon _{3},\delta ,\frac{1}{2}(\delta \pm \varepsilon _{1}\pm \varepsilon _{2}\pm \varepsilon _{3})\}$	$\delta =2\alpha _{1}+3\alpha _{2}+2\alpha _{3}+\alpha _{4}$
$\{\frac{1}{2}(-\delta +\varepsilon _{1}+\varepsilon _{2}+\varepsilon _{3}),\frac{1}{2}(\delta -\varepsilon _{1}-\varepsilon _{2}+\varepsilon _{3}),\varepsilon _{2}-\varepsilon _{3},\varepsilon _{1}-\varepsilon _{2}\}$	$\{\varepsilon _{1},\varepsilon _{2},\varepsilon _{3},\varepsilon _{1}\pm \varepsilon _{2},\varepsilon _{1}\pm \varepsilon _{3},\varepsilon _{2}\pm \varepsilon _{3},\delta ,\frac{1}{2}(\pm \delta +\varepsilon _{1}+\varepsilon _{2}+\varepsilon _{3}),\frac{1}{2}(\delta +\varepsilon _{1}\pm \varepsilon _{2}-\varepsilon _{3}),\frac{1}{2}(\delta -\varepsilon _{1}\pm \varepsilon _{2}+\varepsilon _{3}),\frac{1}{2}(\delta -\varepsilon _{1}+\varepsilon _{2}-\varepsilon _{3}),\frac{1}{2}(\delta +\varepsilon _{1}-\varepsilon _{2}+\varepsilon _{3})\}$	$\frac{1}{2}(\delta +\varepsilon _{1}+\varepsilon _{2}+\varepsilon _{3})=2\alpha _{1}+3\alpha _{2}+2\alpha _{3}+\alpha _{4}$
$\{\varepsilon _{1}-\varepsilon _{2},\frac{1}{2}(\delta -\varepsilon _{1}+\varepsilon _{2}-\varepsilon _{3}),\frac{1}{2}(-\delta +\varepsilon _{1}+\varepsilon _{2}-\varepsilon _{3}),\varepsilon _{3}\}$	$\{\varepsilon _{1},\varepsilon _{2},\varepsilon _{3},\varepsilon _{1}\pm \varepsilon _{2},\varepsilon _{1}\pm \varepsilon _{3},\varepsilon _{2}\pm \varepsilon _{3},\delta ,\frac{1}{2}(\pm \delta +\varepsilon _{1}+\varepsilon _{2}\pm \varepsilon _{3}),\frac{1}{2}(\delta -\varepsilon _{1}+\varepsilon _{2}\pm \varepsilon _{3}),\frac{1}{2}(\delta +\varepsilon _{1}-\varepsilon _{2}\pm \varepsilon _{3})\}$	$\varepsilon _{1}+\varepsilon _{2}=\alpha _{1}+2\alpha _{2}+2\alpha _{3}+2\alpha _{4}$
$\{\frac{1}{2}(\delta +\varepsilon _{1}-\varepsilon _{2}-\varepsilon _{3}),\frac{1}{2}(\delta -\varepsilon _{1}+\varepsilon _{2}+\varepsilon _{3}),\frac{1}{2}(-\delta +\varepsilon _{1}-\varepsilon _{2}+\varepsilon _{3}),\varepsilon _{2}-\varepsilon _{3}\}$	$\{\varepsilon _{1},\varepsilon _{2},\varepsilon _{3},\varepsilon _{1}\pm \varepsilon _{2},\varepsilon _{1}\pm \varepsilon _{3},\varepsilon _{2}\pm \varepsilon _{3},\delta ,\frac{1}{2}(\pm \delta +\varepsilon _{1}\pm \varepsilon _{2}+\varepsilon _{3}),\frac{1}{2}(\pm \delta +\varepsilon _{1}+\varepsilon _{2}-\varepsilon _{3}),\frac{1}{2}(\delta +\varepsilon _{1}-\varepsilon _{2}-\varepsilon _{3}),\frac{1}{2}(\delta -\varepsilon _{1}+\varepsilon _{2}+\varepsilon _{3})$	$\varepsilon _{1}+\varepsilon _{2}=\alpha _{1}+2\alpha _{2}+3\alpha _{3}+2\alpha _{4}$
$\{\delta ,\frac{1}{2}(-\delta +\varepsilon _{1}-\varepsilon _{2}-\varepsilon _{3}),\varepsilon _{3},\varepsilon _{2}-\varepsilon _{3}\}$	$\{\varepsilon _{1},\varepsilon _{2},\varepsilon _{3},\varepsilon _{1}\pm \varepsilon _{2},\varepsilon _{1}\pm \varepsilon _{3},\varepsilon _{2}\pm \varepsilon _{3},\delta ,\frac{1}{2}(\pm \delta +\varepsilon _{1}\pm \varepsilon _{2}\pm \varepsilon _{3})\}$	$\varepsilon _{1}+\varepsilon _{2}=\alpha _{1}+2\alpha _{2}+3\alpha _{3}+2\alpha _{4}$
$\{\delta ,\frac{1}{2}(-\delta -\varepsilon _{1}+\varepsilon _{2}+\varepsilon _{3}),\varepsilon _{1}-\varepsilon _{2},\varepsilon _{2}-\varepsilon _{3}\}$	$\{\varepsilon _{1},\varepsilon _{2},\varepsilon _{3},\varepsilon _{1}\pm \varepsilon _{2},\varepsilon _{1}\pm \varepsilon _{3},\varepsilon _{2}\pm \varepsilon _{3},\delta ,\frac{1}{2}(\pm \delta +\varepsilon _{1}+\varepsilon _{2}\pm \varepsilon _{3}),\frac{1}{2}(\pm \delta -\varepsilon _{1}+\varepsilon _{2}+\varepsilon _{3}),\frac{1}{2}(\pm \delta +\varepsilon _{1}-\varepsilon _{2}+\varepsilon _{3})\}$	$\varepsilon _{1}+\varepsilon _{2}=2\alpha _{1}+4\alpha _{2}+3\alpha _{3}+2\alpha _{4}$
- **4.** *G*(3) The Lie superalgebra $\mathfrak {g}=G(3)$ has the root system $\Phi =\Phi _{\bar{0}}\cup \Phi _{\bar{1}}$ such that $\Phi _{\bar{0}}=\{\pm 2\delta ,\varepsilon _{i}-\varepsilon _{j},\pm \varepsilon _{i}:1\le i\ne j\le 3\}$ and $\Phi _{\bar{1}}=\{\pm \delta \pm \varepsilon _{i},\pm \delta :1\le i\le 3\}$ where $\{\delta ,\varepsilon _{1,}\varepsilon _{2},\varepsilon _{3}\}$ are elements of $(\bigoplus _{i}\mathbb {C}\varepsilon _{i}\oplus \mathbb {C}\delta )/\mathbb {C}(\varepsilon _{1}+\varepsilon _{2}+\varepsilon _{3})$ such that $(\delta ,\delta )=2$, $(\varepsilon _{i},\varepsilon _{j})=1-3\delta _{ij}$, and $(\delta ,\varepsilon _{i})=0$. According to [7, Subsection 5.2], there are four conjugacy classes of systems of simple roots which we list in Table 4.
- **5.** *F*(4) The Lie superalgebra $\mathfrak {g}=F(4)$ has the root system $\Phi =\Phi _{\bar{0}}\cup \Phi _{\bar{1}}$ such that $\Phi _{\bar{0}}=\{\pm \delta ,\pm \varepsilon _{i}\pm \varepsilon _{j},\pm \varepsilon _{i}:i\ne j,i,j=1,2,3\}$ and $\Phi _{\bar{1}}=\{\frac{1}{2}(\pm \delta \pm \varepsilon _{1}\pm \varepsilon _{2}\pm \varepsilon _{3})\}$, where $\{\delta ,\varepsilon _{1,}\varepsilon _{2},\varepsilon _{3}\}$ is an orthogonal basis of $\mathbb {R}\Phi$ such that $(\delta ,\delta )=-6$ and $(\varepsilon _{i},\varepsilon _{j})=2\delta _{ij}$. According to [7, Subsection 6.4], there are six conjugacy classes of systems of simple roots which we list in Table 5.

From above, we know that the highest root depends on the choice of simple roots. In this paper, we define a good prime *p* as below.

#### Definition 5

A prime *p* is said to be *good* for $\mathfrak {g}$ if it is greater than all coefficients $a_{i}$ of $\alpha _{i}$ in the highest root of every system of positive roots $\Phi ^{+}$, and to be *bad* for $\mathfrak {g}$ otherwise.

Thus if $\mathfrak {g}$ is of type *A*(*m*, *n*), then all primes are good; if $\mathfrak {g}$ is of type *B*(*m*, *n*), *C*(*n*), *D*(*m*, *n*) or $D(2,1;\alpha )$, then 2 is bad; if $\mathfrak {g}=G(3)$ or *F*(4), then 2 and 3 are bad.

### Cocharacters Associated to Nilpotent Elements

Let $G_{\bar{0}}$ be a reductive algebraic group over $\mathbb {K}$ defined as in Table 1 so that the adjoint action of $G_{\bar{0}}$ on $\mathfrak {g}$. Recall that any homomorphism of algebraic groups $\tau :\mathbb {K}^{\times }\rightarrow G_{\bar{0}}$ defines a grading $\mathfrak {g}=\bigoplus _{j\in \mathbb {Z}}\mathfrak {g}(j;\tau )$ such that

2.1 $$\begin{aligned} \mathfrak {g}(j;\tau )=\{x\in \mathfrak {g}:\textrm{Ad}(\tau (t))(x)=t^{j}x\text { for all }t\in \mathbb {K}^{\times }\}. \end{aligned}$$

A *parabolic subgroup* *P* of $G_{\bar{0}}$ is a subgroup containing a maximal connected solvable algebraic subgroup of $G_{\bar{0}}$. By [10, Theorem 30.2], any parabolic subgroup *P* of $G_{\bar{0}}$ has a Levi decomposition $P=LV$ where *L* is a *Levi factor*. Throughout this paper, we call a Levi factor of a parabolic subgroup of $G_{\bar{0}}$ a *Levi subgroup* *of* $G_{\bar{0}}$. Let *L* be a Levi subgroup of $G_{\bar{0}}$. A nilpotent element $e\in \mathfrak {g}_{\bar{0}}$ is called *distinguished* in $\textrm{Lie}(L)$ if each torus contained in $L^{e}$ is also contained in the centre *Z*(*L*) of *L*. According to [16, Section 2], every nilpotent element in $\mathfrak {g}_{\bar{0}}$ is distinguished in the Lie algebra of some Levi subgroup of $G_{\bar{0}}$.

#### Definition 6

[12, Definition 5.3]Let $e\in \mathfrak {g}_{\bar{0}}$ be nilpotent. A cocharacter $\tau :\mathbb {K}^{\times }\rightarrow G_{\bar{0}}$ is called *associated to e* if $e\in \mathfrak {g}(2;\tau )$ and there exists a Levi subgroup *L* of $G_{\bar{0}}$ such that *e* is distinguished in *L* and $\textrm{im}(\tau )\subseteq [L,L]$.

By [12, Lemma 5.3], cocharacters associated to *e* do exist if $\textrm{char}(\mathbb {K})$ is good for $G_{\bar{0}}$, and two cocharacters associated to *e* are conjugate under the identity component $(G_{\bar{0}}^{e})^{\circ }$ of $G_{\bar{0}}^{e}$.

#### Theorem 7

[21, Theorem 3.1] Let $\mathfrak {g}=\mathfrak {g}_{\bar{0}}\oplus \mathfrak {g}_{\bar{1}}$ be one of the basic classical Lie superalgebras in Table 2 with $p>3$ if $\mathfrak {g}=D(2,1;\alpha )$ and $p>15$ if $\mathfrak {g}=G(3)$ or *F*(4). Let $e\in \mathfrak {g}_{\bar{0}}$ be nilpotent. Then the cocharacter $\tau$ associated to *e* defines a $\mathbb {Z}$-grading $\mathfrak {g}=\bigoplus _{j\in \mathbb {Z}}\mathfrak {g}(j;\tau )$ such that

2.2 $$\begin{aligned} e\in \mathfrak {g}(2;\tau )\text { and }\mathfrak {g}^{e}=\bigoplus _{j\in \mathbb {Z}}\mathfrak {g}^{e}(j;\tau )\text { where }\mathfrak {g}^{e}(j;\tau )=\mathfrak {g}^{e}\cap \mathfrak {g}(j;\tau ); \end{aligned}$$

2.3 $$\begin{aligned} \mathfrak {g}^{e}(j;\tau )=0\text { for all }j<0\text { and }\dim \mathfrak {g}^{e}=\dim \mathfrak {g}(0;\tau )+\dim \mathfrak {g}(1;\tau ). \end{aligned}$$

Given a nilpotent element $e\in \mathfrak {g}_{\bar{0}}$, we fix the associated cocharacter $\tau$ as follows. Let $\mathfrak {h}$ be a Cartan subalgebra of $\mathfrak {g}$. Recall that the non-degenerate supersymmetric invariant even bilinear form $(\cdot ,\cdot )$ on $\mathfrak {g}$ restricts to a non-degenerate symmetric bilinear form on $\mathfrak {h}$. This allows us to identify $\mathfrak {h}\cong \mathfrak {h}^{*}$ and obtain a symmetric bilinear form $(\cdot ,\cdot )$ on $\mathfrak {h}^{*}$. For $\alpha ,\beta \in \mathfrak {h}^{*}$, we define

2.4 $$\begin{aligned} \langle \beta ,\alpha \rangle =\left\{ \begin{array}{ll} \frac{2(\alpha ,\beta )}{(\alpha ,\alpha )} &{} \text {if }(\alpha ,\alpha )\ne 0;\\ -1 &{} \text {if }(\alpha ,\alpha )=0\text { and }(\alpha ,\beta )\ne 0;\\ 0 &{} \text {if }(\alpha ,\alpha )=(\alpha ,\beta )=0 \end{array}\right.  \end{aligned}$$

as in [4, Section 2.3]. Fix a maximal torus *T* of $G_{\bar{0}}$. Then, we consider a cocharacter $\tau :\mathbb {K}^{\times }\rightarrow G_{\bar{0}}$ associated to *e* in a similar way to [16, Section 6] such that $\tau \left( t\right) =\prod _{i=1}^{s}h_{\alpha _{i}}(t^{c_{i}})$ where $c_{i}$ is defined in Sect. 1. Note that $h_{\alpha _{i}}(t)\in T$ is determined by $h_{\alpha _{i}}(t)\cdot e_{\alpha }=t^{\langle \alpha ,\alpha _{i}\rangle }e_{\alpha }$ for all $t\in \mathbb {K}^{\times }$.

#### Lemma 8

Let $\alpha \in \Phi$. If $e_{\alpha }\in \mathfrak {g}_{\mathbb {Z}}(j;\textrm{ad}h)$, then $e_{\alpha }\in \mathfrak {g}(j;\tau )$.

#### Proof

Suppose $e_{\alpha }\in \mathfrak {g}_{\mathbb {Z}}(j;\textrm{ad}h)$, we know that

$$\begin{aligned} [h,e_{\alpha }]=[\sum _{i=1}^{s}c_{i}h_{i},e_{\alpha }]=\sum _{i=1}^{s}c_{i}[h_{i},e_{\alpha }]=\sum _{i=1}^{s}c_{i}\langle \alpha ,\alpha _{i}\rangle e_{\alpha }. \end{aligned}$$

Hence, we have that $\sum _{i=1}^{s}c_{i}\langle \alpha ,\alpha _{i}\rangle =j$. We also have that

$$\begin{aligned} \tau \left( t\right) \cdot e_{\alpha }=\prod _{i=1}^{s}h_{\alpha _{i}}(t^{c_{i}})\cdot e_{\alpha }=\prod _{i=1}^{s}t^{\langle \alpha ,\alpha _{i}\rangle }e_{\alpha }=t^{\sum _{i=1}^{s}\langle \alpha ,\alpha _{i}\rangle }e_{\alpha }\in \mathfrak {g}(j;\tau ). \end{aligned}$$

Therefore, we obtain that $e_{\alpha }\in \mathfrak {g}(j;\tau )$, as required.$\square$

From now on let us write $\mathfrak {g}(j)$ for $\mathfrak {g}(j;\tau )$ and $\mathfrak {g}^{e}(j)$ for $\mathfrak {g}^{e}(j;\tau )$. We also denote by $\mathfrak {g}_{\bar{i}}^{e}(j)=\mathfrak {g}_{\bar{i}}\cap \mathfrak {g}^{e}(j)$.

## Centralizer and Centre of Centralizer of Nilpotent Elements in Lie Superalgebras of Type *A*(*m*, *n*)

### Centralizer of Nilpotent even Elements in Lie Superalgebras *A*(*m*, *n*)

In this subsection, we recall the centralizer of a nilpotent even element *e* in a Lie superalgebra of type *A*(*m*, *n*) which is analogous to the Lie algebra case in [12, Section 3]. For $\mathfrak {g}\mathfrak {l}(m|n)$ this was done in [9, Subsection 3.2] for a field of zero characteristic and the construction is identical for a field of prime characteristic by [21, Subsection 3.2].

Let $V=V_{\bar{0}}\oplus V_{\bar{1}}$ be a finite-dimensional vector superspace over $\mathbb {K}$ such that $\dim V_{\bar{0}}=m$ and $\dim V_{\bar{1}}=n$. Let $\bar{\mathfrak {g}}=\bar{\mathfrak {g}}_{\bar{0}}\oplus \bar{\mathfrak {g}}_{\bar{1}}=\mathfrak {g}\mathfrak {l}(V)=\mathfrak {g}\mathfrak {l}(m|n)$ and $e\in \bar{\mathfrak {g}}_{\bar{0}}$ be nilpotent. Note that the nilpotent orbits in $\bar{\mathfrak {g}}_{\bar{0}}$ are parametrized by the partitions of (*m*|*n*). Let $\lambda$ be a partition of (*m*|*n*) such that

3.1 $$\begin{aligned} \lambda =(p_{1},\dots ,p_{r}\mid q_{1},\dots ,q_{s}) \end{aligned}$$

where $p_{1}\ge \dots \ge p_{r},q_{1}\ge \dots \ge q_{s}$, $\sum _{i=1}^{r}p_{i}=m$ and $\sum _{i=1}^{s}q_{i}=n$. By rearranging the order of numbers in Eq. 3.1, we write

3.2 $$\begin{aligned} \lambda =(\lambda _{1},\dots ,\lambda _{r+s}) \end{aligned}$$

such that $\lambda _{1}\ge \dots \ge \lambda _{r+s}$. For $i=1,\dots ,r+s$, we define $\bar{i}\in \{\bar{0},\bar{1}\}$ such that for $c\in \mathbb {Z}$, we have that $\left| \{i:\lambda _{i}=c,\bar{i}=\bar{0}\}\right| =\left| \{j:p_{j}=c\}\right|$, $\left| \{i:\lambda _{i}=c,\bar{i}=\bar{1}\}\right| =\left| \{j:q_{j}=c\}\right|$ and if $\lambda _{i}=\lambda _{j}$, $\bar{i}=\bar{0},\bar{j}=\bar{1}$, then $i<j$. i.e. $\sum _{\bar{i}=\bar{0}}\lambda _{i}=m$ and $\sum _{\bar{i}=\bar{1}}\lambda _{i}=n$.

Recall that $\bar{\mathfrak {g}}=\textrm{End}(V_{\bar{0}}\oplus V_{\bar{1}})$ where $\bar{\mathfrak {g}}_{\bar{0}}=\textrm{End}(V_{\bar{0}})\oplus \textrm{End}(V_{\bar{1}})$ and $\bar{\mathfrak {g}}_{\bar{1}}=\textrm{Hom}(V_{\bar{0}},V_{\bar{1}})\oplus \textrm{Hom}(V_{\bar{1}},V_{\bar{0}})$. Then there exist $v_{1},\dots ,v_{r+s}\in V$ such that $V_{\bar{0}}=\langle e^{k}v_{i}:0\le k\le \lambda _{i}-1,\bar{i}=\bar{0}\rangle$, $V_{\bar{1}}=\langle e^{k}v_{i}:0\le k\le \lambda _{i}-1,\bar{i}=\bar{1}\rangle$ and $e^{\lambda _{i}}v_{i}=0$ for $1\le i\le r+s$ by [12, Section 3]. According to [21, Subsection 3.2], the following elements

3.3 $$\begin{aligned} \left\{ \xi _{i}^{j,k}:1\le i,j\le r+s\text { and }\max \{\lambda _{j}-\lambda _{i},0\}\le k\le \lambda _{j}-1\right\} \end{aligned}$$

form a basis for $\bar{\mathfrak {g}}^{e}$ where $\xi _{i}^{j,k}(v_{t})=\delta _{it}e^{k}v_{j}$.

#### Example 9

Suppose $\bar{\mathfrak {g}}=\bar{\mathfrak {g}}_{\bar{0}}\oplus \bar{\mathfrak {g}}_{\bar{1}}=\mathfrak {g}\mathfrak {l}(7|3)=\mathfrak {g}\mathfrak {l}(V)$. Let $e\in \bar{\mathfrak {g}}_{\bar{0}}$ be a nilpotent element that is parametrized by the partition $\lambda =(5,2|3)$. Rewrite $\lambda$ using the notation defined in Eq. 3.2, we have that $\lambda _{1}=5,\lambda _{2}=3$ and $\lambda _{3}=2$. Denote by $J_{\lambda _{i}}$ the $(\lambda _{i}\times \lambda _{i})$-matrix where the $(j,j+1)$ entries with $1\le j<\lambda _{i}$ are equal to 1 and all remaining entries are equal to zero. Then the order of basis for *V* is rearranged to be $(e^{4}v_{1},e^{3}v_{1},e^{2}v_{1},ev_{1},v_{1},e^{2}v_{2},ev_{2},v_{2},ev_{3},v_{3})$ such that $v_{1},v_{3}\in V_{\bar{0}}$, $v_{2}\in V_{\bar{1}}$ and with respect to this basis *e* has block form

$$\begin{aligned} \begin{pmatrix}J_{\lambda _{1}} &{} 0 &{} 0\\ 0 &{} J_{\lambda _{2}} &{} 0\\ 0 &{} 0 &{} J_{\lambda _{3}} \end{pmatrix}. \end{aligned}$$

**Fig. 1:**
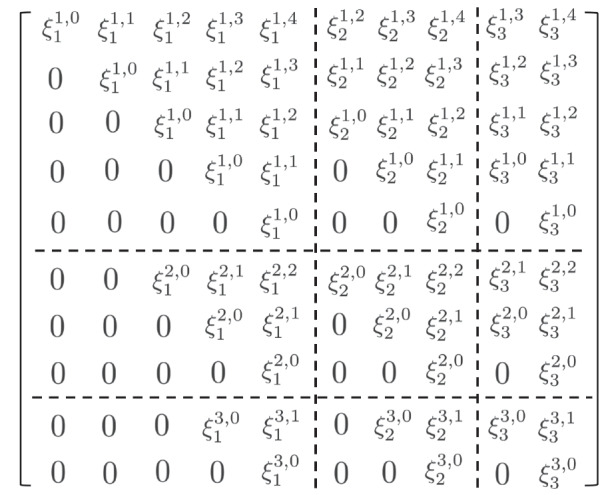
$$\xi _{i}^{j,k}$$

In Fig. 1, we write elements $\xi _{i}^{j,k}\in \bar{\mathfrak {g}}^{e}$ in block matrices with respect to the basis for *V*.

Now let $\mathfrak {g}=\mathfrak {g}_{\bar{0}}\oplus \mathfrak {g}_{\bar{1}}=\mathfrak {s}\mathfrak {l}(V)=\mathfrak {s}\mathfrak {l}(m|n)$. Based on the above example, in order to determine $\mathfrak {g}^{e}$, we need to let $\sum _{i=1}^{r+s}(-1)^{\bar{i}}\lambda _{i}\xi _{i}^{i,0}=0$. Note that if $\textrm{char}(\mathbb {K})=p$ divides $\lambda _{i}$ for all $1\le i\le r+s$, we have that $\sum _{i=1}^{r+s}(-1)^{\bar{i}}\lambda _{i}\xi _{i}^{i,0}=0$. Hence, we deduce that $\dim \mathfrak {g}^{e}=\dim \bar{\mathfrak {g}}^{e}$ and the construction of $\bar{\mathfrak {g}}^{e}$ is identical to $\mathfrak {g}^{e}$ for this case. However, if there exist some $\lambda _{i}$ which is not divisible by *p*, we have that $\dim \mathfrak {g}^{e}=\dim \bar{\mathfrak {g}}^{e}-1$ as $\sum _{i=1}^{r+s}(-1)^{\bar{i}}\lambda _{i}\xi _{i}^{i,0}$ has to be zero. Thus the diagonal matrices $\xi _{i}^{i,0}$ are not in $\mathfrak {g}^{e}$ and a basis for $\mathfrak {g}^{e}$ is of the form

$$\begin{aligned} \left\{ \xi _{i}^{j,k}:1\le i\ne j\!\le \! r\!+\!s\text { and }\max \{\lambda _{j}\!-\!\lambda _{i},0\}\!\le \! k \!\le \!\lambda _{j}\!-\!1\right\} \cup \left\{ \xi _{i}^{i,k}:0<k\!\le \!\lambda _{i}\!-\!1\right\} \end{aligned}$$

$$\begin{aligned} \cup \left\{ \frac{\lambda _{i+1}}{p^{a}}\xi _{i}^{i,0}\!-\!(\!-\!1)^{\bar{i}}\frac{\lambda _{i}}{p^{a}}\xi _{i+1}^{i+1,0}:1\!\le \! i\!< \!r+s,a\text { is maximal such that }p^{a}\text { divides both }\lambda _{i}\text { and }\lambda _{i+1}\right\} . \end{aligned}$$

Note that when $m=n$, we consider $\mathfrak {g}=\mathfrak {g}_{\bar{0}}\oplus \mathfrak {g}_{\bar{1}}=\mathfrak {p}\mathfrak {s}\mathfrak {l}(n|n)$ instead of $\mathfrak {s}\mathfrak {l}(n|n)$. Let $e\in \mathfrak {g}_{\bar{0}}$ be nilpotent. By abuse of notation, we use the same letter *e* for its lift in $\mathfrak {s}\mathfrak {l}(n|n)$.

#### Lemma 10

When *p* does not divide *n*, we have that $\dim \mathfrak {g}^{e}=\dim \mathfrak {s}\mathfrak {l}(n|n)^{e}-1$.

#### Proof

Let $\phi :\mathfrak {s}\mathfrak {l}(n|n)\rightarrow \mathfrak {g}$ be the quotient map. For $x=x_{\bar{0}}+x_{\bar{1}}$ such that $x+\mathfrak {z}(\mathfrak {s}\mathfrak {l}(n|n))\in \mathfrak {g}^{e}$, we have that $[e,x]\in \mathfrak {z}(\mathfrak {s}\mathfrak {l}(n|n))$. Since $[e,x]=[e,x_{\bar{0}}]+[e,x_{\bar{1}}]$, then $[e,x]\in \mathfrak {z}(\mathfrak {s}\mathfrak {l}(n|n))$ implies that $[e,x_{\bar{1}}]=0$ and $[e,x_{\bar{0}}]\in \mathfrak {z}(\mathfrak {s}\mathfrak {l}(n|n))$. When *p* does not divide *n*, this is impossible since $[e,x_{\bar{0}}]\in [\mathfrak {g}_{\bar{0}},\mathfrak {g}_{\bar{0}}]=\mathfrak {s}\mathfrak {l}_{n}(\mathbb {K})\oplus \mathfrak {s}\mathfrak {l}_{n}(\mathbb {K})$ and $\mathfrak {z}(\mathfrak {s}\mathfrak {l}(n|n))\notin \mathfrak {s}\mathfrak {l}_{n}(\mathbb {K})\oplus \mathfrak {s}\mathfrak {l}_{n}(\mathbb {K})$. Hence, we have that $\phi ^{e}:\mathfrak {s}\mathfrak {l}(n|n)^{e}\rightarrow \mathfrak {g}^{e}$ is surjective and $\textrm{ker}(\phi ^{e})=\mathfrak {z}(\mathfrak {s}\mathfrak {l}(n|n))$. Therefore, we deduce that $\dim \mathfrak {g}^{e}=\dim \mathfrak {s}\mathfrak {l}(n|n)^{e}-1$. $\square$

Since $\mathfrak {z}(\mathfrak {s}\mathfrak {l}(n|n))\subseteq \mathfrak {s}\mathfrak {l}(n|n)^{e}$ and $\phi$ sends $\mathfrak {z}(\mathfrak {s}\mathfrak {l}(n|n))$ to zero, we can write a basis for $\mathfrak {g}^{e}$ as

3.4 $$\begin{aligned} \begin{array}{c}\left\{ {\xi }_{i}^{j,k}:1\le i\ne j\le r+s \textrm{andmax}\left\{ {\lambda }_{j}-{\lambda }_{i},0\right\} \le k\le {\lambda }_{j}-1\right\}  \end{array} \end{aligned}$$

$$\begin{aligned} \cup \left\{ {\xi }_{i}^{j,k}:1\le i\le r+s,0<k\le {\lambda }_{i}-1\right\} \end{aligned}$$

$$\begin{aligned} \cup \left\{ \! \frac{\lambda _{i+1}}{p^{a}}\xi _{i}^{i,0}\!-\!(\!-\!1)^{\bar{i}}\frac{\lambda _{i}}{p^{a}}\xi _{i+1}^{i+1,0}\!:1\!<\!i\!<\!r\!+\!s,a\text { is maximal such that }p^{a}\text { divides both }\lambda _{i}\text { and }\lambda _{i+1}\right\} . \end{aligned}$$

#### Remark 11

Note that when *p* divides *n*, then $\mathfrak {z}(\mathfrak {s}\mathfrak {l}(n|n))\subseteq \mathfrak {s}\mathfrak {l}_{n}(\mathbb {K})\oplus \mathfrak {s}\mathfrak {l}_{n}(\mathbb {K})$ and $\phi ^{e}:\mathfrak {s}\mathfrak {l}(n|n)^{e}\rightarrow \mathfrak {g}^{e}$ is not surjective. In this paper, we rule out this case when considering $\mathfrak {p}\mathfrak {s}\mathfrak {l}(n|n)^{e}$ and $\mathfrak {z}(\mathfrak {p}\mathfrak {s}\mathfrak {l}(n|n)^{e})$.

### Centre of Centralizer of Nilpotent even Elements in Lie Superalgebras *A*(*m*, *n*)

The centre of centralizer of a nilpotent even element *e* in $\mathfrak {g}\mathfrak {l}(m|n)$ was done for a field of zero characteristic in [6, Subsection 3.3], we prove that the structure is identical over our field $\mathbb {K}$ in the following theorem.

#### Theorem 12

Let $\bar{\mathfrak {g}}=\bar{\mathfrak {g}}_{\bar{0}}\oplus \bar{\mathfrak {g}}_{\bar{1}}=\mathfrak {g}\mathfrak {l}(m|n)$. Let $e\in \bar{\mathfrak {g}}_{\bar{0}}$ be nilpotent and $\lambda =(\lambda _{1},\dots ,\lambda _{r+s})$ be the partition with respect to *e* defined as in Eq. 3.2. Then $\mathfrak {z}(\bar{\mathfrak {g}}^{e})=\langle e^{0},e,\dots ,e^{\lambda _{1}-1}\rangle$ where the first element, $e^{0}$, is the identity matrix.

#### Proof

Let us denote by $I_{m|0}\in \bar{\mathfrak {g}}^{e}$ be the $(m+n)\times (m+n)$ matrix such that

$$\begin{aligned} I_{m|0}=\begin{pmatrix}I_{m} &{} 0_{m\times n}\\ 0_{n\times m} &{} 0_{n\times n} \end{pmatrix} \end{aligned}$$

where $I_{m}$ is the $m\times m$ identity matrix and $0_{m\times n},0_{n\times m},0_{n\times n}$ are matrices with zero entries. For any $x\in \mathfrak {z}(\bar{\mathfrak {g}}^{e})$, we have that $[I_{m|0},x]=0$. Hence, we obtain that $\mathfrak {z}(\bar{\mathfrak {g}}^{e})\subseteq \bar{\mathfrak {g}}^{I_{m|0}}$. Let $$y=\begin{pmatrix}A &{} B\\ C &{} D \end{pmatrix}\in \bar{\mathfrak {g}}$$, we compute that $[I_{m|0},y]=0$ if and only if $B=C=0$. Thus, we deduce that $y\in \bar{\mathfrak {g}}_{\bar{0}}$ and $\bar{\mathfrak {g}}^{I_{m|0}}=\bar{\mathfrak {g}}_{\bar{0}}$. Therefore, we have that $\mathfrak {z}(\bar{\mathfrak {g}}^{e})\subseteq \bar{\mathfrak {g}}_{\bar{0}}$.

Observe that $\bar{\mathfrak {g}}$ can be viewed as a vector space that is isomorphic to $\mathfrak {g}\mathfrak {l}_{m+n}(\mathbb {K})$. We also have that $\bar{\mathfrak {g}}^{e}$ is isomorphic to $\mathfrak {g}\mathfrak {l}_{m+n}(\mathbb {K})^{e}$ as a vector space because $[e,x]=[e,x']$ for $x\in \bar{\mathfrak {g}}$ and $x'\in \mathfrak {g}\mathfrak {l}_{m+n}(\mathbb {K})$. Let us denote

$$\begin{aligned} \mathfrak {z}(\mathfrak {g}\mathfrak {l}_{m+n}(\mathbb {K})^{e})_{0}\!=\!\mathfrak {z}(\mathfrak {g}\mathfrak {l}_{m+n}(\mathbb {K})^{e})\cap \left\{ \! \begin{pmatrix}A' &{} 0\\ 0 &{} D' \end{pmatrix}\in \mathfrak {g}\mathfrak {l}_{m+n}(\mathbb {K}):A'\!\in \!\mathfrak {g}\mathfrak {l}_{m}(\mathbb {K})\text { and }D'\!\in \!\mathfrak {g}\mathfrak {l}_{n}(\mathbb {K})\right\} , \end{aligned}$$

$$\begin{aligned} \text {and }\mathfrak {z}(\mathfrak {g}\mathfrak {l}_{m+n}(\mathbb {K})^{e})_{1}\!=\!\mathfrak {z}(\mathfrak {g}\mathfrak {l}_{m+n}(\mathbb {K})^{e})\cap \left\{ \begin{pmatrix}0 &{} B'\\ C' &{} 0 \end{pmatrix}:B'\text { is a }m\!\times \! n\text { matrix, }C'\text { is a }n\!\times \! m\text { matrix}\right\} . \end{aligned}$$

Let $\mathfrak {z}(\bar{\mathfrak {g}}^{e})=\mathfrak {z}(\bar{\mathfrak {g}}^{e})_{\bar{0}}\oplus \mathfrak {z}(\bar{\mathfrak {g}}^{e})_{\bar{1}}.$ Note that $\mathfrak {z}(\bar{\mathfrak {g}}^{e})_{\bar{0}}=\mathfrak {z}(\mathfrak {g}\mathfrak {l}_{m+n}(\mathbb {K})^{e})_{0}$ by definition. Since $\mathfrak {z}(\bar{\mathfrak {g}}^{e})\subseteq \bar{\mathfrak {g}}_{\bar{0}}$, we have that $\mathfrak {z}(\bar{\mathfrak {g}}^{e})_{\bar{1}}=0$. Using a similar argument as in the first part of this proof, we also obtain that $\mathfrak {z}(\mathfrak {g}\mathfrak {l}_{m+n}(\mathbb {K})^{e})_{1}=0$. By [16, Section 4], we know that $\mathfrak {z}(\mathfrak {g}\mathfrak {l}_{m+n}(\mathbb {K})^{e})=\langle I,e,\dots ,e^{\lambda _{1}-1}\rangle$. Therefore, we obtain that $\mathfrak {z}(\bar{\mathfrak {g}}^{e})=\mathfrak {z}(\bar{\mathfrak {g}}^{e})_{\bar{0}}=\mathfrak {z}(\mathfrak {g}\mathfrak {l}_{m+n}(\mathbb {K})^{e})=\mathfrak {z}(\mathfrak {g}\mathfrak {l}_{m+n}(\mathbb {K})^{e})_{0}=\langle I,e,\dots ,e^{\lambda _{1}-1}\rangle$.$\square$

Next, we look at the centre of centralizer of a nilpotent element $e\in \mathfrak {s}\mathfrak {l}(m|n)_{\bar{0}}$ when $m\ne n$ and if $m=n$, we consider $\mathfrak {p}\mathfrak {s}\mathfrak {l}(n|n)$ instead.

#### Theorem 13

Let $\mathfrak {g}=\mathfrak {g}_{\bar{0}}\oplus \mathfrak {g}_{\bar{1}}=\mathfrak {s}\mathfrak {l}(m|n)$ for $m\ne n$. Let $e\in \mathfrak {g}_{\bar{0}}$ be nilpotent and $\lambda =(\lambda _{1},\dots ,\lambda _{r+s})$ be the partition with respect to *e* defined as in Eq. 3.2. If $\textrm{char}(\mathbb {K})=p$ divides $\lambda _{i}$ for all $1\le i\le r+s$, then $\mathfrak {z}(\mathfrak {g}^{e})=\langle I,e,e^{2},\dots ,e^{\lambda _{1}-1}\rangle$; If there exists some $\lambda _{i}$ which is not divisible by *p*, then $\mathfrak {z}(\mathfrak {g}^{e})=\langle e,e^{2},\dots ,e^{\lambda _{1}-1}\rangle$.

#### Proof

We again write $\bar{\mathfrak {g}}=\bar{\mathfrak {g}}_{\bar{0}}\oplus \bar{\mathfrak {g}}_{\bar{1}}=\mathfrak {g}\mathfrak {l}(m|n)$. When $m\ne n$. If $\textrm{char}(\mathbb {K})=p$ divides $\lambda _{i}$ for all $1\le i\le r+s$, then $\mathfrak {g}^{e}=\bar{\mathfrak {g}}^{e}$ and thus by Theorem 12, we have that $\mathfrak {z}(\mathfrak {g}^{e})=\langle I,e,e^{2},\dots ,e^{\lambda _{1}-1}\rangle$. If there exists some $\lambda _{i}$ which is not divisible by *p*, then $\bar{\mathfrak {g}}^{e}\subseteq \mathfrak {g}^{e}\oplus \mathbb {K}I$ and thus $\mathfrak {z}(\bar{\mathfrak {g}}^{e})\subseteq \mathfrak {z}(\mathfrak {g}^{e})\oplus \mathbb {K}I$. For an element $x\in \mathfrak {z}(\mathfrak {g}^{e})$, observe that $[x,I]=0$ and $[x,y]=0$ for all $y\in \mathfrak {g}^{e}$. Hence, we deduce that $[x,y]=0$ for all $y\in \bar{\mathfrak {g}}^{e}$ and thus $x\in \mathfrak {z}(\bar{\mathfrak {g}}^{e})$. This implies that $\mathfrak {z}(\mathfrak {g}^{e})\subseteq \mathfrak {z}(\bar{\mathfrak {g}}^{e})$. Therefore, we obtain that a basis for $\mathfrak {z}(\mathfrak {g}^{e})$ contains all basis vectors of $\mathfrak {z}(\bar{\mathfrak {g}}^{e})$ except the identity matrix *I*.$\square$

In the remaining part of this subsection, let $\mathfrak {g}=\mathfrak {g}_{\bar{0}}\oplus \mathfrak {g}_{\bar{1}}=\mathfrak {p}\mathfrak {s}\mathfrak {l}(n|n)$ and let $e\in \mathfrak {g}_{\bar{0}}$ be nilpotent. Below, we give a basis of $\mathfrak {z}(\mathfrak {g}^{e})$. Note that a basis of $\mathfrak {z}(\mathfrak {g}_{\mathbb {C}}^{e})$ is given in [8, Subsection 4.2] and we apply a similar argument in the following theorem. By abuse of notation, we use the same letter *e* for its lift in $\mathfrak {s}\mathfrak {l}(n|n)$.

#### Theorem 14

Let $\mathfrak {g}=\mathfrak {g}_{\bar{0}}\oplus \mathfrak {g}_{\bar{1}}$ be a Lie superalgebra of type $\mathfrak {p}\mathfrak {s}\mathfrak {l}(n|n)$ over $\mathbb {K}$ such that $\textrm{char}(\mathbb {K})=p$ does not divide *n*. Let $\lambda =(\lambda _{1},\dots ,\lambda _{r+s})$ be a partition of (*n*|*n*) defined as in Eq. 3.2 and suppose $e\in \mathfrak {g}_{\bar{0}}$ is a nilpotent element with Jordan type $\lambda$. Then $\mathfrak {z}(\mathfrak {g}^{e})=\langle e,e^{2},\dots ,e^{\lambda _{1}-1}\rangle$.

#### Proof

From Eq. 3.4, we know that an element $x\in \mathfrak {g}^{e}$ is of the form

$$\begin{aligned} x=&\underset{\max \{\lambda _{j}-\lambda _{i},0\}\le k\le \lambda _{j}-1}{\sum\limits_{1\le i\ne j\le r+s}}a_{i,j,k}\xi _{i}^{j,k}+\underset{1\le i\le r+s}{\sum\limits_{0< k \le \lambda _{i}-1}}b_{i,k}\xi _{i}^{i,k}\nonumber\\ &+\sum _{1 < i < r+s} c_{i,0}\left( \frac{\lambda _{i+1}}{p^{a}}\xi _{i}^{i,0}-(-1)^{\bar{i}}\frac{\lambda _{i}}{p^{a}}\xi _{i+1}^{i+1,0}\right) \end{aligned}$$

where $$a_{i,j,k},b_{i,k},c_{i,0}\in \mathbb {K}$$.

Now assume $x\in \mathfrak {z}(\mathfrak {g}^{e})$. We divide our analysis into two cases.

1. When $r+s\ge 3$. Let us denote by $\mathfrak {h}$ the Cartan subalgebra of $\mathfrak {g}$. Then elements $\frac{\lambda _{j^{'}}}{p^{a}}\xi _{i^{'}}^{i^{'},0}-(-1)^{\bar{i^{'}}}\frac{\lambda _{i^{'}}}{p^{a}}\xi _{j^{'}}^{j^{'},0}\in \mathfrak {h}$ for $i^{'}\ne j^{'}$ where *a* is maximal such that $p^{a}$ divides both $\lambda _{i^{'}}$ and $\lambda _{j^{'}}$. Thus, we have that $[x,\frac{\lambda _{j^{'}}}{p^{a}}\xi _{i^{'}}^{i^{'},0}-(-1)^{\bar{i^{'}}}\frac{\lambda _{i^{'}}}{p^{a}}\xi _{j^{'}}^{j^{'},0}]\in \left\langle \sum _{1\le i\le r+s}\xi _{i}^{i,0}\right\rangle$. We have that
  3.5 $$\begin{aligned} {}[x,\frac{\lambda _{j^{'}}}{p^{a}}\xi _{i^{'}}^{i^{'},0}-(-1)^{\bar{i^{'}}}\frac{\lambda _{i^{'}}}{p^{a}}\xi _{j^{'}}^{j^{'},0}] =&\frac{\lambda _{j^{'}}}{p^{a}}\left( \sum _{j,k}a_{i^{'},j,k}\xi _{i^{'}}^{j,k}-\sum _{i,k}a_{i,i^{'},k}\xi _{i}^{i^{'},k}\right) \nonumber \\ &\!-\!(\!-\!1)^{\bar{i^{'}}}\frac{\lambda _{i^{'}}}{p^{a}}\left( \sum _{j,k}a_{j^{'},j,k}\xi _{j^{'}}^{j,k}\!-\!\sum _{i,k}a_{i,j^{'},k}\xi _{i}^{j^{'},k}\right) . \end{aligned}$$
  In order to obtain a summand of $\xi _{i}^{i,0}$ with nonzero coefficients for any *i* from Eq. 3.5, we need $k=0$, $j=i^{'}$ (resp. $k=0$, $i=i^{'}$) in the first (resp. second) summand and we have $a_{i^{'},i^{'},0}\xi _{i^{'}}^{i^{'},0}-a_{i^{'},i^{'},0}\xi _{i^{'}}^{i^{'},0}=0$. Similarly, we have $a_{j^{'},j^{'},0}\xi _{j^{'}}^{j^{'},0}-a_{j^{'},j^{'},0}\xi _{j^{'}}^{j^{'},0}=0$ in the third and fourth summands. Hence, we deduce that $[x,\frac{\lambda _{j^{'}}}{p^{a}}\xi _{i^{'}}^{i^{'},0}-(-1)^{\bar{i^{'}}}\frac{\lambda _{i^{'}}}{p^{a}}\xi _{j^{'}}^{j^{'},0}]=0$. This gives us $a_{i,j,k}=0$ for all $1\le i\ne j\le r+s$ and thus the element *x* is reduced to the form
  $$\begin{aligned} x=\sum _{\begin{array}{c} 0<k\le \lambda _{i}-1\\ 1\le i\le r+s \end{array} }b_{i,k}\xi _{i}^{i,k}+\sum _{1<i<r+s}c_{i,0}\left( \frac{\lambda _{i+1}}{p^{a}}\xi _{i}^{i,0}-(-1)^{\bar{i}}\frac{\lambda _{i}}{p^{a}}\xi _{i+1}^{i+1,0}\right) . \end{aligned}$$
  Next, we consider an element $\xi _{i^{'}}^{j^{'},0}\in \mathfrak {g}^{e}$ for some $i^{'}<j^{'}$. Using a similar argument as above to $[x,\xi _{i^{'}}^{j^{'},0}]$, we obtain that $c_{i,0}=0$ for $1<i<r+s$ and $b_{j^{'},k}=b_{i^{'},k}$ for all $1\le i^{'}\ne j^{'}\le r+s$ and $0<k\le \lambda _{1}-1$. By the definition of $\xi _{i}^{i,k}$ in Eq. 3.3, we have that $e^{k}=\sum _{i=1}^{r+s}\xi _{i}^{i,k}$. Therefore, we deduce that $x\in \mathfrak {z}(\mathfrak {g}^{e})$ if and only if $x\in \langle e,\dots ,e^{\lambda _{1}-1}\rangle$.
2. When $r+s=2$, i.e. $\lambda =(\lambda _{1}|\lambda _{2})$ and $\lambda _{1}=\lambda _{2}=n>1$. Then an element $y\in \mathfrak {g}^{e}$ can be written as $y=\sum _{1\le i\ne j\le 2,0\le k\le n-1}a_{i,j,k}\xi _{i}^{j,k}+\sum _{0<k\le n-1,1\le i\le 2}b_{i,k}\xi _{i}^{i,k}$ for $a_{i,j,k},b_{i,k}\in \mathbb {K}$. Now assume $y\in \mathfrak {z}(\mathfrak {g}^{e})$. Applying a similar argument as in case 1 to $[y,\xi _{1}^{1,1}]$ and $[y,\xi _{2}^{1,0}]$, we deduce that $a_{1,2,k}=a_{2,1,k}=0$ for $0\le k\le n-1$ and $b_{1,k}=b_{2,k}$ for $0<k\le n-1$. In conclusion, we have that $\mathfrak {z}(\mathfrak {g}^{e})=\langle e,\dots ,e^{\lambda _{1}-1}\rangle$.$\square$

## Centralizer and Centre of Centralizer of Nilpotent Elements in Ortho-Symplectic Lie Superalgebras

### Centralizer of Nilpotent even Elements in Ortho-Symplectic Lie Superalgebras

Let $V=V_{\bar{0}}\oplus V_{\bar{1}}$ be a finite-dimensional vector superspace over $\mathbb {K}$ such that $\dim V_{\bar{0}}=m$ and $\dim V_{\bar{1}}=2n$. Let *B* be a non-degenerate even supersymmetric bilinear form on *V*, that is, $V_{\bar{0}}$ and $V_{\bar{1}}$ are orthogonal, the restriction of *B* to $V_{\bar{0}}$ is symmetric, and the restriction of *B* to $V_{\bar{1}}$ is skew-symmetric. Then the ortho-symplectic Lie superalgebra $\mathfrak {g}=\mathfrak {o}\mathfrak {s}\mathfrak {p}(V)=\mathfrak {o}\mathfrak {s}\mathfrak {p}(m|2n)$ is defined to be $\mathfrak {o}\mathfrak {s}\mathfrak {p}(V)=\mathfrak {g}_{\bar{0}}\oplus \mathfrak {g}_{\bar{1}}$ where

$$ \mathfrak {g}_{\bar{i}}=\{x\in \mathfrak {g}\mathfrak {l}(V)_{\bar{i}}:B(x(v),w)=-(-1)^{\bar{i}\bar{x}}B(v,x(w))\} $$

for homogeneous $v,w\in V$ and $\bar{i}\in \mathbb {Z}_{2}$.

Let $e\in \mathfrak {g}_{\bar{0}}$ be nilpotent with the corresponding Jordan type $\lambda$ such that $\lambda =(\lambda _{1},\dots ,\lambda _{r+s})$ is defined in a similar way to Eq. 3.2 where $\sum _{\bar{i}=\bar{0}}\lambda _{i}=m$ and $\sum _{\bar{i}=\bar{1}}\lambda _{i}=2n$. In this subsection, we describe the structure of the centralizer $\mathfrak {g}^{e}$. In case of zero characteristic, a basis for $\mathfrak {g}_{\mathbb {C}}^{e}$ was given in [6, Subsection 4.5], in particular, it contains all elements in the set *S* in Eq. 4.1.

According to [6, Subsection 4.5], there exists an involution $i\mapsto i^{*}$ on the set $\{1,\dots ,r+s\}$ such that $i^{*}\in \{i-1,i,i+1\}$ for all $1\le i\le r+s$, $\lambda _{i}=\lambda _{i^{*}}$ and $\bar{i^{*}}=\bar{i}$. Note that $i=i^{*}$ if $\bar{i}=\bar{0}$ and $(-1)^{\lambda _{i}}=-1$ or if $\bar{i}=\bar{1}$ and $(-1)^{\lambda _{i}}=1$. Recall that basis elements $\xi _{i}^{j,k}$ for $\mathfrak {g}\mathfrak {l}(m|2n)^{e}$ are defined in Eq. 3.3. Let $S=S_{0}\cup S_{1}\cup S_{2}$ be a subset of $\mathfrak {g}$ given by

4.1 $$\begin{aligned} S_{0}=\{\xi _{i}^{i^{*},\lambda _{i}-1-k}:0\le k\le \lambda _{i}-1,\bar{i}=\bar{0},k\text { is odd}\}, \end{aligned}$$

$$ S_{1}=\{\xi _{i}^{i^{*},\lambda _{i}-1-k}:0\le k\le \lambda _{i}-1,\bar{i}=\bar{1},k\text { is even}\}, $$

$$ \text {and }S_{2}\!=\!\{\xi _{i}^{j,\lambda _{j}-1-k}\!+\!\varepsilon _{i,j,k}\xi _{j^{*}}^{i^{*},\lambda _{i}-1-k}:0\!\le \! k\!\le \!\min \{\lambda _{i},\lambda _{j}\}\!-\!1,i^{*}\!\ne \! j,\varepsilon _{i,j,k}\!\in \!\{\pm 1\}\}. $$

We have that $\varepsilon _{i,j,k}=(-1)^{\lambda _{j}-k-\bar{x}\bar{i}}\theta _{j}\theta _{i}$ where $\bar{x}\in \{\bar{0},\bar{1}\}$ is the parity of $\xi _{i}^{j,\lambda _{j}-1-k}$ and $\theta _{j},\theta _{i}\in \{\pm 1\}$ can be determined explicitly by [12, Section 3.2]. Note that an element $\xi _{i}^{j,\lambda _{j}-1-k}+\varepsilon _{i,j,k}\xi _{j^{*}}^{i^{*},\lambda _{i}-1-k}$ lies in $\mathfrak {g}_{\bar{0}}^{e}$ (resp. $\mathfrak {g}_{\bar{1}}^{e}$ ) if $\bar{i}=\bar{j}$ (resp. $\bar{i}\ne \bar{j}$).

#### Theorem 15

We have that *S* is a basis for $\mathfrak {g}^{e}$.

#### Proof

We know that $e=\sum _{i=1}^{r+s}\xi _{i}^{i,1}$. Commutators between *e* and elements in *S* over $\mathbb {Q}$ have been calculated explicitly in [6, Subsection 4.5], they all equal to zeros thus they must also be zeros in case of $\mathbb {K}$. Hence, we deduce that *S* is a subset of $\mathfrak {g}^{e}$. Based on the conditions on indices *i*, *j*, *k* and that $\xi _{i}^{j,\lambda _{j}-1-k}$ are linearly independent in $\mathfrak {g}\mathfrak {l}(m|2n)$, for $c_{i,i^{*},k},c_{i,j,k}\in \mathbb {K}$, $\sum c_{i,i^{*},k}\xi _{i}^{i^{*},\lambda _{i}-1-k}+\sum _{i^{*}\ne j}c_{i,j,k}(\xi _{i}^{j,\lambda _{j}-1-k}+\varepsilon _{i,j,k}\xi _{j^{*}}^{i^{*},\lambda _{i}-1-k})=0$ implies that $c_{i,i^{*},k}=c_{i,j,k}=0$. Thus, we have that elements $\xi _{i}^{i^{*},\lambda _{i}-1-k}$ and $\xi _{i}^{j,\lambda _{j}-1-k}+\varepsilon _{i,j,k}\xi _{j^{*}}^{i^{*},\lambda _{i}-1-k}$ in *S* are linearly independent. By [6, Proposition 20], we know that $|S|=\dim \mathfrak {g}_{\mathbb {C}}(0)+\dim \mathfrak {g}_{\mathbb {C}}(1)$. According to Lemma 8, we have that $\dim \mathfrak {g}_{\mathbb {C}}(j)=\dim \mathfrak {g}(j)$ for all $j\in \mathbb {Z}$ and thus $|S|=\dim \mathfrak {g}(0)+\dim \mathfrak {g}(1)$. Furthermore, recall that $\dim \mathfrak {g}^{e}=\dim \mathfrak {g}(0)+\dim \mathfrak {g}(1)$ by Theorem 7. Therefore, we deduce that $|S|=\dim \mathfrak {g}^{e}$ and *S* is a basis for $\mathfrak {g}^{e}$.$\square$

### Centre of Centralizer of Nilpotent even Elements in Ortho-Symplectic Lie Superalgebras

A basis of $\mathfrak {z}(\mathfrak {g}_{\mathbb {C}}^{e})$ is given in [6, Section 4] and we apply a similar argument to $\mathfrak {g}$ in this subsection. In particular, we prove Theorem 16 which gives a basis of $\mathfrak {z}(\mathfrak {g}^{e})$, though note that some explicit calculations are omitted as they are the same as in [6, Section 4].

Before we start the proof, we need the following notation. In this subsection, we use an alternative notation for $\lambda$ and rewrite

4.2 $$\begin{aligned} \lambda =(\lambda _{1},\dots ,\lambda _{a},\lambda _{a+1},\lambda _{-(a+1)},\dots ,\lambda _{b},\lambda _{-b}) \end{aligned}$$

where $\lambda _{1},\dots ,\lambda _{a}$ are the parts with odd multiplicity, $\lambda _{1}>\lambda _{2}>\dots >\lambda _{a}$ and $\lambda _{a+1}=\lambda _{-(a+1)}\ge \dots \ge \lambda _{b}=\lambda _{-b}$. We define $\bar{i}\in \{\bar{0},\bar{1}\}$ such that for $c\in \mathbb {Z}$, we have $\left| \{i:\lambda _{i}=c,\bar{i}=\bar{0}\}\right| =\left| \{j:p_{j}=c\}\right|$ and $\left| \{i:\lambda _{i}=c,\bar{i}=\bar{1}\}\right| =\left| \{j:q_{j}=c\}\right|$ for some *j*. Then there exists $\{v_{1},\dots ,v_{a},v_{a+1},v_{-(a+1)},\dots ,v_{b},v_{-b}\}\in V$ such that our vector superspace *V* can be decomposed as $V=V_{1}\oplus V_{2}$ where $V_{1}=\langle e^{j}v_{i}:1\le i\le a\rangle$ and $V_{2}=\langle e^{j}v_{i},e^{j}v_{-i}:a+1\le i\le b\rangle$. Let $\mathfrak {g}_{1}=\mathfrak {osp}(V_{1})$ and $\mathfrak {g}_{2}=\mathfrak {o}\mathfrak {s}\mathfrak {p}(V_{2})$, we define $\bar{\mathfrak {g}}=\mathfrak {g}_{1}\oplus \mathfrak {g}_{2}$. Then a nilpotent element $e\in \mathfrak {g}_{\bar{0}}$ can be viewed as $e=e_{1}+e_{2}$ where $e_{i}\in \mathfrak {g}_{i}$ and the Jordan type of $e_{1}$ (resp. $e_{2}$) is $(\lambda _{1},\dots ,\lambda _{a})$ (resp. $(\lambda _{a+1},\lambda _{-(a+1)},\dots ,\lambda _{b},\lambda _{-b})$). We have that $\mathfrak {z}(\mathfrak {g}^{e})\subseteq \mathfrak {z}(\mathfrak {g}_{1}^{e_{1}})\oplus \mathfrak {z}(\mathfrak {g}_{2}^{e_{2}})$ by [6, Subsection 4.8].

#### Theorem 16

Let $A=\{e^{l}:l\text { is odd and }0\le l\le \min \{\lambda _{1},\lambda _{a+1}\}-1\}$. Then *A* is a basis for $\mathfrak {z}(\mathfrak {g}^{e})$ except for the following two cases:

- If $a\ge 3$, $\lambda _{2}>\lambda _{a+1}$ and $\bar{i}=\bar{0}$ for $i=1,2$ or $a=2,\bar{i}\ne \bar{j}$ for $(i,j)=(1,2)$, then, we have that $A\cup \{\xi _{1}^{2,\lambda _{2}-1}-\xi _{2}^{1,\lambda _{1}-1}\}$ is a basis for $\mathfrak {z}(\mathfrak {g}^{e})$;
- If $\lambda _{1}<\lambda _{a+1}$, $\lambda _{a+1}>\lambda _{a+2}$, $\bar{i}=\bar{0}$ for $i=a+1$ and $\lambda _{a+1}$ is odd, we have that $A\cup \{\xi _{a+1}^{a+1,\lambda _{a+1}-1}-\xi _{-(a+1)}^{-(a+1),\lambda _{a+1}-1}\}$ is a basis for $\mathfrak {z}(\mathfrak {g}^{e})$.

#### Proof

Step 1: Determine $$\mathfrak {z}(\mathfrak {g}_{1}^{e_{1}})$$.

According to Subsection 4.1, we know that an element $x_{1}\in \mathfrak {z}(\mathfrak {g}_{1}^{e_{1}})$ can be written as

$$\begin{aligned} x_{1}= \underset{1\le i \le a}{\sum\limits_{0\le k\le \lambda _{i}-1,\lambda _{i}-k\text { is even}}}c_{i,k}\xi _{i}^{i,\lambda _{i}-1-k}+\underset{1 \le i < j \le a}{\sum\limits_{0 \le k \le \lambda_{j}-1}}c_{i,j,k}(\xi _{i}^{j,\lambda _{j}-1-k}+\varepsilon _{i,j,k}\xi _{j}^{i,\lambda _{i}-1-k}) \end{aligned} $$

where $$c_{i,k},c_{i,j,k}\in \mathbb {K}$$.

Suppose $a\ge 3$. For an element $\xi _{t}^{t,1}\in \mathfrak {g}^{e}$, we know that $[\xi _{t}^{t,1},x_{1}]=0$ for $1\le t\le a$. By calculating this we obtain that $c_{i,j,k}=0$ for $1\le k\le \lambda _{j}-1$. For $1\le t<h\le a$, the commutator $[\xi _{t}^{h,0}+\varepsilon _{t,h,\lambda _{h}-1}\xi _{h}^{t,\lambda _{t}-\lambda _{h}},x_{1}]=0$ implies that $c_{t,k}=c_{h,k}$ for all $1\le t<h\le a$ and $c_{i,j,0}=0$ for all $1\le i<j\le a$ except when $\bar{i}=\bar{0}$ for $i=1,2$. Hence, we deduce that $\mathfrak {z}(\mathfrak {g}_{1}^{e_{1}})=\langle \sum _{i=1}^{a}\xi _{i}^{i,l}:l\text { is odd and }1\le l\le \lambda _{1}-1\rangle$ except when $\bar{i}=\bar{0}$ for $i=1,2$, in which case, we have that $\xi _{1}^{2,\lambda _{2}-1}-\xi _{2}^{1,\lambda _{1}-1}$ commutes with all basis elements in $\mathfrak {g}^{e}$.

Applying a similar argument as above to the case in which $a=2$, we obtain that $c_{1,2,k}=0$ for $1\le k\le 2n-1$, $c_{1,k}=c_{2,k}$ for all *k* and $\xi _{1}^{2,\lambda _{2}-1}-\xi _{2}^{1,\lambda _{1}-1}$ commutes with all basis elements in $\mathfrak {g}^{e}$.

Therefore, we have that $\mathfrak {z}(\mathfrak {g}_{1}^{e_{1}})=\langle e_{1}^{l}:l\text { is odd and }1\le l\le \lambda _{1}-1\rangle$ except when $a\ge 3$ and $\bar{i}=\bar{0}$ for $i=1,2$ or $a=2,\bar{i}\ne \bar{j}$ for $(i,j)=(1,2)$, in which case, we have that $\mathfrak {z}(\mathfrak {g}_{1}^{e_{1}})=\langle e_{1}^{l}:l\text { is odd and }1\le l\le \lambda _{1}-1\rangle \oplus \langle \xi _{1}^{2,\lambda _{2}-1}-\xi _{2}^{1,\lambda _{1}-1}\rangle$.

Step 2: Determine $$\mathfrak {z}(\mathfrak {g}_{2}^{e_{2}})$$.

Based on arguments in [6, Subsection 4.6], we have that $\mathfrak {z}(\mathfrak {g}_{2}^{e_{2}})\subseteq (\mathfrak {g}_{2}^{e_{2}})^{\mathfrak {h}_{2}^{e_{2}}}$ where $\mathfrak {h}_{2}$ is a Cartan subalgebra of $\mathfrak {g}_{2}$ and $\mathfrak {h}_{2}^{e_{2}}=\langle h_{i}=\xi _{i}^{i,0}-\xi _{-i}^{-i,0}:a+1\le i\le b\rangle$. By computing commutators between an element $h\in \mathfrak {h}_{2}^{e_{2}}$ and basis elements of $\mathfrak {g}_{2}^{e_{2}}$, we deduce that $(\mathfrak {g}_{2}^{e_{2}})^{\mathfrak {h}_{2}^{e_{2}}}=\langle \xi _{j}^{j,\lambda _{j}-1-k}+\varepsilon _{j,j,k}\xi _{-j}^{-j,\lambda _{j}-1-k}:a+1\le j\le b\rangle$. Now let $x_{2}\in \mathfrak {z}(\mathfrak {g}_{2}^{e_{2}})$ such that $x_{2}=\sum _{j,k}c_{j,k}(\xi _{j}^{j,\lambda _{j}-1-k}+\varepsilon _{j,j,k}\xi _{-j}^{-j,\lambda _{j}-1-k})$ where $c_{j,k}\in \mathbb {K}$. Note that for $a+1\le i\le b$, an element $\xi _{i}^{-i,0}\in \mathfrak {g}_{2}^{e_{2}}$ (resp. $\xi _{i}^{-i,1}\in \mathfrak {g}_{2}^{e_{2}}$) if $\lambda _{i}$ is even and $\bar{i}=\bar{0}$ (resp. $\lambda _{i}$ is odd and $\bar{i}=\bar{1}$), then $[\xi _{i}^{-i,0},x_{2}]=0$ (resp. $[\xi _{i}^{-i,1},x_{2}]=0$) implies that $c_{j,k}=0$ if $\lambda _{j}-k$ is odd and $k\ne 0$. For $\lambda _{j}-k$ is odd and $k=0$, if there exists some $\lambda _{i}$ such that $a+1\le i\le b$ and $\lambda _{i}\ge \lambda _{j}$, by considering $[\xi _{i}^{j,0}+\varepsilon _{i,j,\lambda _{j}-1}\xi _{-j}^{-i,\lambda _{i}-\lambda _{j}},x_{2}]=0$ we have that $c_{j,0}=0$. Hence, we deduce that $\mathfrak {z}(\mathfrak {g}_{2}^{e_{2}})\subseteq \langle \xi _{j}^{j,\lambda _{j}-1-k}+\xi _{-j}^{-j,\lambda _{j}-1-k}:a+1\le j\le b,\lambda _{j}-k\text { is even}\rangle$ except when $\lambda _{a+1}$ is odd, $\lambda _{a+1}>\lambda _{i}$ for all $a+2\le |i|\le b$ and $\overline{a+1}=\bar{0}$, in which case $\xi _{a+1}^{a+1,\lambda _{a+1}-1}-\xi _{-(a+1)}^{-(a+1),\lambda _{a+1}-1}$ commutes with all basis elements in $\mathfrak {g}_{2}^{e_{2}}$. Furthermore, for $a+1\le i<t\le b$, the commutator $[\xi _{i}^{t,0}+\varepsilon _{i,t,\lambda _{t}-1}\xi _{-t}^{-i,\lambda _{i}-\lambda _{t}},x_{2}]=0$ gives us $c_{i,k}=c_{t,k}$ whenever all $\lambda _{i}-k$ and $\lambda _{t}-k$ are even.

Therefore, we have that $\mathfrak {z}(\mathfrak {g}_{2}^{e_{2}})=\langle e_{2}^{l}:l\text { is odd and }1\le l\le \lambda _{a+1}-1\rangle$ except when $\lambda _{a+1}$ is odd, $\lambda _{a+1}>\lambda _{i}$ for all $a+2\le |i|\le b$ and $\overline{a+1}=\bar{0}$, in which case, we have that $\mathfrak {z}(\mathfrak {g}_{2}^{e_{2}})=\langle e_{2}^{l}:l\text { is odd and }1\le l\le \lambda _{a+1}-1\rangle \oplus \langle \xi _{a+1}^{a+1,\lambda _{a+1}-1}-\xi _{-(a+1)}^{-(a+1),\lambda _{a+1}-1}\rangle$.

Step 3: Determine $$\mathfrak {z}(\mathfrak {g}^{e})$$.

We first consider the case when $\mathfrak {z}(\mathfrak {g}_{1}^{e_{1}})=\langle e_{1}^{l}:l\text { is odd and }1\le l\le \lambda _{1}-1\rangle$ and $\mathfrak {z}(\mathfrak {g}_{2}^{e_{2}})=\langle e_{2}^{l}:l\text { is odd and }1\le l\le \lambda _{a+1}-1\rangle$. Since $\mathfrak {z}(\mathfrak {g}^{e})\subseteq \mathfrak {z}(\mathfrak {g}_{1}^{e_{1}})\oplus \mathfrak {z}(\mathfrak {g}_{2}^{e_{2}})$, an element $x\in \mathfrak {z}(\mathfrak {g}^{e})$ is of the form

$$ x=\sum _{l\text { is odd;}l=1}^{\lambda _{1}-1}a_{l}\left( \sum _{t=1}^{a}\xi _{t}^{t,l}\right) +\sum _{l\text { is odd;}k=1}^{\lambda _{a+1}-1}b_{l}\left( \sum _{t=a+1}^{b}(\xi _{t}^{t,l}+\xi _{-t}^{-t,l})\right) $$

for $a_{l},b_{l}\in \mathbb {K}$. If $\lambda _{1}\ge \lambda _{a+1}$ (resp. $\lambda _{1}<\lambda _{a+1}$), by letting $[\xi _{1}^{a+1,0}+\varepsilon _{1,a+1,\lambda _{a+1}-1}\xi _{-(a+1)}^{1,\lambda _{1}-\lambda _{a+1}},x]=0$, (resp. $[\xi _{1}^{a+1,\lambda _{a+1}-\lambda _{1}}+\varepsilon _{1,a+1,\lambda _{1}-1}\xi _{-(a+1)}^{1,0},x]=0$) we obtain that $a_{l}=b_{l}$ for all odd *l*. Hence, in this case, we deduce that *A* is a basis for $\mathfrak {z}(\mathfrak {g}^{e})$.

It remains to consider special cases.

(i) When $\xi _{1}^{2,\lambda _{2}-1}-\xi _{2}^{1,\lambda _{1}-1}\in \mathfrak {z}(\mathfrak {g}_{1}^{e_{1}})$ and $\xi _{a+1}^{a+1,\lambda _{a+1}-1}-\xi _{-(a+1)}^{-(a+1),\lambda _{a+1}-1}\notin \mathfrak {z}(\mathfrak {g}_{2}^{e_{2}})$, an element $y\in \mathfrak {z}(\mathfrak {g}^{e})$ is of the form $y=x+c_{1,2,0}(\xi _{1}^{2,\lambda _{2}-1}-\xi _{2}^{1,\lambda _{1}-1})$. Applying a similar argument as above to *y* gives us $a_{l}=b_{l}$ for all odd *l* and $c_{1,2,0}=0$ except for $\lambda _{2}>\lambda _{a+1}$, in which case $\xi _{1}^{2,\lambda _{2}-1}-\xi _{2}^{1,\lambda _{1}-1}$ commutes with all basis elements of $\mathfrak {g}^{e}$.
(ii) When $\xi _{1}^{2,\lambda _{2}-1}-\xi _{2}^{1,\lambda _{1}-1}\notin \mathfrak {z}(\mathfrak {g}_{1}^{e_{1}})$ and $\xi _{a+1}^{a+1,\lambda _{a+1}-1}-\xi _{-(a+1)}^{-(a+1),\lambda _{a+1}-1}\in \mathfrak {z}(\mathfrak {g}_{2}^{e_{2}})$, an element $z\in \mathfrak {z}(\mathfrak {g}^{e})$ is of the form $z=x+c_{a+1,a+1,0}(\xi _{a+1}^{a+1,\lambda _{a+1}-1}-\xi _{-(a+1)}^{-(a+1),\lambda _{a+1}-1})$. Applying a similar argument as above to *z* gives us $a_{l}=b_{l}$ for all odd *l* and $c_{a+1,a+1,0}=0$ except for $\lambda _{1}<\lambda _{a+1}$, in which case $\xi _{a+1}^{a+1,\lambda _{a+1}-1}-\xi _{-(a+1)}^{-(a+1),\lambda _{a+1}-1}$ commutes with all basis elements of $\mathfrak {g}^{e}$.
(iii) When $\xi _{1}^{2,\lambda _{2}-1}-\xi _{2}^{1,\lambda _{1}-1}\in \mathfrak {z}(\mathfrak {g}_{1}^{e_{1}})$ and $\xi _{a+1}^{a+1,\lambda _{a+1}-1}-\xi _{-(a+1)}^{-(a+1),\lambda _{a+1}-1}\in \mathfrak {z}(\mathfrak {g}_{2}^{e_{2}})$, applying a similar argument as above, we also have that $\mathfrak {z}(\mathfrak {g}^{e})=\langle A\rangle \oplus \langle \xi _{1}^{2,\lambda _{2}-1}-\xi _{2}^{1,\lambda _{1}-1}\rangle$ for $\lambda _{2}>\lambda _{a+1}$ and $\mathfrak {z}(\mathfrak {g}^{e})=\langle A\rangle \oplus \langle \xi _{a+1}^{a+1,\lambda _{a+1}-1}-\xi _{-(a+1)}^{-(a+1),\lambda _{a+1}-1}\rangle$ for $\lambda _{1}<\lambda _{a+1}$.$\square$

## Centralizer and Centre of Centralizer of Nilpotent Elements in Exceptional Lie Superalgebras

In this section, we fix the following notation. For an element $x=x_{\bar{0}}+x_{\bar{1}}\in \mathfrak {g}$, we have that $[e,x]=[e,x_{\bar{0}}]+[e,x_{\bar{1}}]$ where $[e,x_{\bar{0}}]\in \mathfrak {g}_{\bar{0}}$ and $[e,x_{\bar{1}}]\in \mathfrak {g}_{\bar{1}}$. This implies that $\mathfrak {g}^{e}=\mathfrak {g}_{\bar{0}}^{e}\oplus \mathfrak {g}_{\bar{1}}^{e}$. Similarly, let us denote $\mathfrak {z}(\mathfrak {g}^{e})=\mathfrak {z}_{\bar{0}}\oplus \mathfrak {z}_{\bar{1}}$ and $\mathfrak {z}_{\bar{i}}(j)=\mathfrak {z}_{\bar{i}}\cap \mathfrak {g}(j)$. We often work with $\mathfrak {sl}_{2}(\mathbb {K})$ so we fix notation *E*, *H*, *F* for its basis elements where

$$\begin{aligned} E=\begin{pmatrix}0 &{} 1\\ 0 &{} 0 \end{pmatrix},H=\begin{pmatrix}1 &{} 0\\ 0 &{} -1 \end{pmatrix},F=\begin{pmatrix}0 &{} 0\\ 1 &{} 0 \end{pmatrix}. \end{aligned}$$

### Centralizer of even Nilpotent Elements in $D(2,1;\alpha )$ and its Centre

The Lie superalgebras $D(2,1;\alpha )$ with $\alpha \in \mathbb {K}\backslash \{0,-1\}$ form a one-parameter family of seventeen-dimensional Lie superalgebras. Note that $D(2,1;\alpha )$ is also denoted by $\Gamma (\sigma _{1},\sigma _{2},\sigma _{3})$ in [19] where $\sigma _{1},\sigma _{2},\sigma _{3}\in \mathbb {K}\backslash \{0,1\}$ and $\sigma _{1}+\sigma _{2}+\sigma _{3}=0$. According to [17, Lemma 5.5.16], if there is another triple $(\sigma _{1}^{'},\sigma _{2}^{'},\sigma _{3}^{'})$ such that $\sigma _{1}^{'}+\sigma _{2}^{'}+\sigma _{3}^{'}=0$ and $\Gamma (\sigma _{1}^{'},\sigma _{2}^{'},\sigma _{3}^{'})\cong \Gamma (\sigma _{1},\sigma _{2},\sigma _{3})$, then there exist a permutation $\rho$ of $\{1,2,3\}$ and a nonzero complex number *c* such that $\sigma _{i}^{'}$ can be obtained by $\sigma _{i}^{'}=c\sigma _{\rho (i)}$. For any $\alpha \in \mathbb {K}\backslash \{0,-1\}$, we have $D(2,1;\alpha )=\Gamma (1+\alpha ,-1,-\alpha )\cong \Gamma (-\alpha ,-1,1+\alpha )\cong \Gamma (\frac{1+\alpha }{\alpha },-1,-\frac{1}{\alpha })$.

Let $\mathfrak {g}=\mathfrak {g}_{\bar{0}}\oplus \mathfrak {g}_{\bar{1}}=D(2,1;\alpha )$ with $\alpha \in \mathbb {K}\backslash \{0,-1\}$. By definition the even part is $\mathfrak {g}_{\bar{0}}=\mathfrak {sl}_{2}(\mathbb {K})\oplus \mathfrak {sl}_{2}(\mathbb {K})\oplus \mathfrak {sl}_{2}(\mathbb {K})$ and the odd part is $\mathfrak {g}_{\bar{1}}=V_{1}\otimes V_{2}\otimes V_{3}$ where $V_{i}$, $i=1,2,3$ is the standard two-dimensional $\mathfrak {sl}_{2}(\mathbb {K})$-module for the *i*th summand in $\mathfrak {g}_{\bar{0}}$ with basis elements $v_{1}^{i}=(1,0)^{t}$ and $v_{-1}^{i}=(0,1)^{t}$. Note that the map $\mathfrak {g}_{\bar{0}}\times \mathfrak {g}_{\bar{0}}\rightarrow \mathfrak {g}_{\bar{0}}$ is a Lie bracket and the Lie superbracket $[\cdot ,\cdot ]:\mathfrak {g}_{\bar{0}}\times \mathfrak {g}_{\bar{1}}\rightarrow \mathfrak {g}_{\bar{1}}$ is defined by $[x_{1}\oplus x_{2}\oplus x_{3},v_{i}^{1}\otimes v_{j}^{2}\otimes v_{k}^{3}]=x_{1}v_{i}^{1}\otimes v_{j}^{2}\otimes v_{k}^{3}+v_{i}^{1}\otimes x_{2}v_{j}^{2}\otimes v_{k}^{3}+v_{i}^{1}\otimes v_{j}^{2}\otimes x_{3}v_{k}^{3}$ for $x_{1}\oplus x_{2}\oplus x_{3}\in \mathfrak {g}_{\bar{0}}$, $v_{i}^{1}\otimes v_{j}^{2}\otimes v_{k}^{3}\in \mathfrak {g}_{\bar{1}}$. The Lie superbracket $[\cdot ,\cdot ]:\mathfrak {g}_{\bar{1}}\times \mathfrak {g}_{\bar{1}}\rightarrow \mathfrak {g}_{\bar{0}}$ is given for example in [7, Section 4.2] and it depends on $\sigma _{i}$ for $i=1,2,3$.

In this subsection, we denote $E_{1}=(E,0,0)$, $E_{2}=(0,E,0)$ and $E_{3}=(0,0,E)$. Similarly, let $F_{1}=(F,0,0)$, $F_{2}=(0,F,0)$, $F_{3}=(0,0,F)$, $H_{1}=(H,0,0)$, $H_{2}=(0,H,0)$ and $H_{3}=(0,0,H)$. We write $v_{i,j,k}$ for $v_{i}^{1}\otimes v_{j}^{2}\otimes v_{k}^{3}$ where $i,j,k\in \{\pm 1\}$. Then a basis for $\mathfrak {g}_{\bar{0}}$ is $\{E_{i},H_{i},F_{i}:i=1,2,3\}$ and a basis for $\mathfrak {g}_{\bar{1}}$ is $\{v_{i,j,k}:i,j,k=\pm 1\}$.

Since representatives of nilpotent orbits in $\mathfrak {sl}_{2}(\mathbb {K})$ up to conjugation by $\textrm{SL}_{2}(\mathbb {K})$ are 0 and *E*, we have that representatives of nilpotent orbits $e\in \mathfrak {g}_{\bar{0}}$ are $e=0,E_{1},E_{2},E_{3},E_{1}+E_{2},E_{2}+E_{3},E_{1}+E_{3},E_{1}+E_{2}+E_{3}$. We give the cocharacter $\tau$ associated to *e* and basis elements for $\mathfrak {g}^{e}$ and $\mathfrak {z}\left( \mathfrak {g}^{e}\right)$ for $e=0,E_{1},E_{1}+E_{2},E_{1}+E_{2}+E_{3}$ in Table 6. Note that in the second column of Table 6, these $\alpha _{i}=2\beta _{i}$, $i=1,2,3$ are even roots in the root system for $\mathfrak {g}$ as given in Subsection 2.2. After Table 6, we explain our explicit calculation for the case $e=E_{1}+E_{2}+E_{3}$. The cases when $e=0$, $E_{1}$, $E_{1}+E_{2}$ are obtained using a similar approach. Note that analysis for cases $e=E_{2}$, $e=E_{3}$ are similar to $e=E_{1}$ and analysis for cases $e=E_{1}+E_{3}$, $e=E_{2}+E_{3}$ are similar to $e=E_{1}+E_{2}$, for which are omitted in this paper (Table 7).

**Table 6:** $$\tau $$, $$\mathfrak {g}^{e}$$ and $$\mathfrak {z}\left( \mathfrak {g}^{e}\right) $$

Representatives of nilpotent orbits $e\in \mathfrak {g}_{\bar{0}}$	Cocharacter $\tau$	$\mathfrak {g}^{e}=\mathfrak {g}_{\bar{0}}^{e}\oplus \mathfrak {g}_{\bar{1}}^{e}$	$\dim \left( \mathfrak {g}^{e}\right)$	$\mathfrak {z}\left( \mathfrak {g}^{e}\right)$
0	0	$\mathfrak {g}$	17	$\langle e\rangle =0$
$E_{1}$	$h_{\alpha _{1}}(t)$	$\langle E_{1},E_{2},H_{2},F_{2},E_{3},H_{3},F_{3}\rangle \oplus$	11	$\langle e\rangle$
		$$ \langle v_{1,j,k}:j,k=\pm 1\rangle $$		
$E_{1}+E_{2}$	$h_{\alpha _{1}}(t)h_{\alpha _{2}}(t)$	$\langle E_{1},E_{2},E_{3},H_{3},F_{3}\rangle \oplus$	9	$\langle e\rangle$
		$$ \langle v_{1,1,1},v_{1,1,-1},v_{1,-1,1}-$$		
		$$v_{-1,1,1},v_{1,-1,-1}-v_{-1,1,-1}\rangle $$		
$E_{1}+E_{2}+E_{3}$	$h_{\alpha _{1}}(t)h_{\alpha _{2}}(t)h_{\alpha _{3}}(t)$	$\langle E_{1},E_{2},E_{3}\rangle \oplus \langle v_{1,1,1},v_{1,1,-1}-$	6	$\langle e,v_{1,1,1}\rangle$
		$$v_{-1,1,1},v_{1,-1,1}-v_{-1,1,1}\rangle $$		

Now, we give our calculation explicitly for the case $e=E_{1}+E_{2}+E_{3}$. Since $\mathfrak {g}_{\bar{0}}=\mathfrak {s}\mathfrak {l}_{2}(\mathbb {K})\oplus \mathfrak {s}\mathfrak {l}_{2}(\mathbb {K})\oplus \mathfrak {s}\mathfrak {l}_{2}(\mathbb {K})$, we easily compute that $\mathfrak {g}_{\bar{0}}^{e}=\langle E_{1},E_{2},E_{3}\rangle$. To determine $\mathfrak {g}_{\bar{1}}^{e}$, we give the $\tau$-grading on basis elements of $\mathfrak {g}_{\bar{1}}$ in Table 7.

**Table 7:** The $\tau$-grading on basis elements of $\mathfrak {g}_{\bar{1}}$when $e=E_{1}+E_{2}+E_{3}$

$\tau$-grading	basis elements of $\mathfrak {g}_{\bar{1}}$
3	$v_{1,1,1}$
1	$v_{1,1,-1},v_{1,-1,1},v_{-1,1,1}$
$-1$	$v_{1,-1,-1},v_{-1,1,-1},v_{-1,-1,1}$
$-3$	$v_{-1,-1,-1}$

From the above table, we have that $\mathfrak {g}_{\bar{1}}^{e}=\mathfrak {g}_{\bar{1}}^{e}(3)\oplus \mathfrak {g}_{\bar{1}}^{e}(1)\oplus \mathfrak {g}_{\bar{1}}^{e}(-1)\oplus \mathfrak {g}_{\bar{1}}^{e}(-3)$. We know that $[e,\mathfrak {g}_{\bar{1}}(3)]\subseteq \mathfrak {g}_{\bar{1}}(5)$ and since $\mathfrak {g}_{\bar{1}}(5)=0$, we have that $\mathfrak {g}_{\bar{1}}^{e}(3)=\langle v_{1,1,1}\rangle$. In order to determine $\mathfrak {g}_{\bar{1}}^{e}(1)$, we consider $x=a_{1,1,-1}v_{1,1,-1}+a_{1,-1,1}v_{1,-1,1}+a_{-1,1,1}v_{-1,1,1}\in \mathfrak {g}_{\bar{1}}^{e}(1)$ for $a_{1,1,-1}$, $a_{1,-1,1}$, $a_{-1,1,1}\in \mathbb {K}$. Then we compute $[e,x]=(a_{1,1,-1}+a_{1,-1,1}+a_{-1,1,1})v_{1,1,1}$. This equals to zero implies that $a_{1,1,-1}+a_{1,-1,1}+a_{-1,1,1}=0$. Thus, we have that $\mathfrak {g}_{\bar{1}}^{e}(1)=\langle v_{1,1,1},v_{1,1,-1}-v_{-1,1,1},v_{1,-1,1}-v_{-1,1,1}\rangle$. To determine $\mathfrak {g}_{\bar{1}}^{e}(-1)$, we consider $y=a_{1,-1,-1}v_{1,-1,-1}+a_{-1,1,-1}v_{-1,1,-1}+a_{-1,-1,1}v_{-1,-1,1}\in \mathfrak {g}_{\bar{1}}^{e}(-1)$ for $a_{1,-1,-1}$, $a_{-1,1,-1}$, $a_{-1,-1,1}\in \mathbb {K}$. We have that $[e,y]=(a_{-1,1,-1}+a_{1,-1,-1})v_{1,1,-1}+(a_{-1,-1,1}+a_{1,-1,-1})v_{1,-1,1}+(a_{-1,-1,1}+a_{-1,1,-1})v_{-1,1,1},$ this equals to zero implies that $a_{1,-1,-1}=a_{-1,1,-1}=a_{-1,-1,1}=0$. Hence, we deduce that $\mathfrak {g}_{\bar{1}}^{e}(-1)=0$. We also obtain that $\mathfrak {g}_{\bar{1}}^{e}(-3)=0$ since $[e,v_{-1,-1,-1}]=v_{1,-1,-1}+v_{-1,1,-1}+v_{-1,-1,1}\ne 0$. Therefore, we obtain that $\mathfrak {g}^{e}=\langle E_{1},E_{2},E_{3}\rangle \oplus \langle v_{1,1,1},v_{1,1,-1}-v_{-1,1,1},v_{1,-1,1}-v_{-1,1,1}\rangle$.

To determine $\mathfrak {z}(\mathfrak {g}^{e})$, we let $x=b_{1}E_{1}+b_{2}E_{2}+b_{3}E_{3}\in \mathfrak {z}_{\bar{0}}$. Then $[x,v_{1,1,-1}-v_{-1,1,1}]=0$ and $[x,v_{1,-1,1}-v_{-1,1,1}]=0$ provide us with $b_{1}=b_{2}=b_{3}$. Hence, we obtain that $\mathfrak {z}_{\bar{0}}=\langle E_{1}+E_{2}+E_{3}\rangle =\langle e\rangle$. Next, we look at $\mathfrak {z}_{\bar{1}}=\mathfrak {z}_{\bar{1}}(3)\oplus \mathfrak {z}_{\bar{1}}(1)$. Let $z=b_{4}(v_{1,1,-1}-v_{-1,1,1})+b_{5}(v_{1,-1,1}-v_{-1,1,1})\in \mathfrak {z}_{\bar{1}}(3)$ for $b_{4},b_{5}\in \mathbb {K}$. We calculate $[E_{2},z]=b_{5}v_{1,1,1}$ and $[E_{3},z]=b_{4}v_{1,1,1}$, these commutators are equal to zero if and only if $b_{4}=b_{5}=0$. Hence, we deduce that $\mathfrak {z}_{\bar{1}}(1)=0$. Now, we consider $\mathfrak {z}_{\bar{1}}(3)$. Observe that $\mathfrak {g}^{e}(3+j)=0$ for all *j* such that $\mathfrak {g}^{e}(j)\ne 0$. Hence, we have that $[w,v_{1,1,1}]=0$ for all $w\in \mathfrak {g}^{e}(j)\ne 0$ and thus $\mathfrak {z}_{\bar{1}}(3)=\langle v_{1,1,1}\rangle$. Therefore, we obtain that $\mathfrak {z}(\mathfrak {g}^{e})=\langle e,v_{1,1,1}\rangle$.

### Centralizer of even Nilpotent Elements in *G*(3) and its Centre

Let $\mathfrak {g}=\mathfrak {g}_{\bar{0}}\oplus \mathfrak {g}_{\bar{1}}=G(3)$. The explicit description of the Lie superalgebra *G*(3) is given for example in [7, Section 5]. Recall that the even part $\mathfrak {g}_{\bar{0}}$ is a direct sum of Lie algebras $\mathfrak {s}\mathfrak {l}_{2}(\mathbb {K})$ and $G_{2}$. The odd part $\mathfrak {g}_{\bar{1}}=V_{2}\otimes V_{7}$ where $V_{2}=\langle v_{1}=(1,0)^{t},v_{-1}=(0,1)^{t}\rangle$ is the standard two-dimensional representation for $\mathfrak {s}\mathfrak {l}_{2}(\mathbb {K})$ and $V_{7}$ is a seven-dimensional simple representation of $G_{2}$ with a basis $\{e_{3},e_{2},e_{1},e_{0},e_{-1},e_{-2},e_{-3}\}$. Thus $\mathfrak {g}_{\bar{1}}$ has a basis $\{v_{i}\otimes e_{j}:i=\pm 1,j=0,\pm 1,\pm 2,\pm 3\}$, see for example [7, Section 5].

In this subsection, we write the elements of $G_{2}$ with respect to the basis of $V_{7}$ since $G_{2}$ can be viewed as a Lie subalgebra of $\mathfrak {gl}(V_{7})$. In this case $G_{2}=\langle x_{i},y_{i},h_{1},h_{2}:i=1,\dots ,6\rangle$ where

$$\begin{aligned} x_{1}=\begin{pmatrix}0 &{} -1 &{} 0 &{} 0 &{} 0 &{} 0 &{} 0\\ 0 &{} 0 &{} 0 &{} 0 &{} 0 &{} 0 &{} 0\\ 0 &{} 0 &{} 0 &{} 1 &{} 0 &{} 0 &{} 0\\ 0 &{} 0 &{} 0 &{} 0 &{} -2 &{} 0 &{} 0\\ 0 &{} 0 &{} 0 &{} 0 &{} 0 &{} 0 &{} 0\\ 0 &{} 0 &{} 0 &{} 0 &{} 0 &{} 0 &{} 1\\ 0 &{} 0 &{} 0 &{} 0 &{} 0 &{} 0 &{} 0 \end{pmatrix},\ x_{2}=\begin{pmatrix}0 &{} 0 &{} 0 &{} 0 &{} 0 &{} 0 &{} 0\\ 0 &{} 0 &{} 1 &{} 0 &{} 0 &{} 0 &{} 0\\ 0 &{} 0 &{} 0 &{} 0 &{} 0 &{} 0 &{} 0\\ 0 &{} 0 &{} 0 &{} 0 &{} 0 &{} 0 &{} 0\\ 0 &{} 0 &{} 0 &{} 0 &{} 0 &{} -1 &{} 0\\ 0 &{} 0 &{} 0 &{} 0 &{} 0 &{} 0 &{} 0\\ 0 &{} 0 &{} 0 &{} 0 &{} 0 &{} 0 &{} 0 \end{pmatrix}, \end{aligned}$$

$$\begin{aligned} y_{1}=\begin{pmatrix}0 &{} 0 &{} 0 &{} 0 &{} 0 &{} 0 &{} 0\\ -1 &{} 0 &{} 0 &{} 0 &{} 0 &{} 0 &{} 0\\ 0 &{} 0 &{} 0 &{} 0 &{} 0 &{} 0 &{} 0\\ 0 &{} 0 &{} 2 &{} 0 &{} 0 &{} 0 &{} 0\\ 0 &{} 0 &{} 0 &{} -1 &{} 0 &{} 0 &{} 0\\ 0 &{} 0 &{} 0 &{} 0 &{} 0 &{} 0 &{} 0\\ 0 &{} 0 &{} 0 &{} 0 &{} 0 &{} 1 &{} 0 \end{pmatrix},\ y_{2}=\begin{pmatrix}0 &{} 0 &{} 0 &{} 0 &{} 0 &{} 0 &{} 0\\ 0 &{} 0 &{} 0 &{} 0 &{} 0 &{} 0 &{} 0\\ 0 &{} 1 &{} 0 &{} 0 &{} 0 &{} 0 &{} 0\\ 0 &{} 0 &{} 0 &{} 0 &{} 0 &{} 0 &{} 0\\ 0 &{} 0 &{} 0 &{} 0 &{} 0 &{} 0 &{} 0\\ 0 &{} 0 &{} 0 &{} 0 &{} -1 &{} 0 &{} 0\\ 0 &{} 0 &{} 0 &{} 0 &{} 0 &{} 0 &{} 0 \end{pmatrix}, \end{aligned}$$

$$h_{1}=\text {diag}(1,-1,2,0,-2,1,-1)$$, $$h_{2}=\text {diag}(0,1,-1,0,1,-1,0)$$, and $$x_{3}=[x_{1},x_{2}]$$, $$x_{4}=[x_{1},x_{3}]$$, $$x_{5}=[x_{1},x_{4}]$$, $$x_{6}=[x_{5},x_{2}]$$, $$y_{3}=[y_{1},y_{2}]$$, $$y_{4}=[y_{1},y_{3}]$$, $$y_{5}=[y_{1},y_{4}]$$, $$y_{6}=[y_{5},y_{2}]$$.

**Table 8:** Cocharacters and $\mathfrak {g}^{e}$ for $\mathfrak {g}=G(3)$

Representatives of nilpotent orbits $e\in \mathfrak {g}_{\bar{0}}$	Cocharacter $\tau$	$\mathfrak {g}^{e}$	$\dim \mathfrak {g}^{e}$	$\mathfrak {z}\left( \mathfrak {g}^{e}\right)$
0	0	$\mathfrak {g}$	31	0
$x_{2}$	$h_{\alpha _{2}}(t)$	$\langle E,H,F,2h_{1}+3h_{2},x_{2},x_{3},x_{6},y_{1},y_{5},x_{4},y_{4}\rangle \oplus \langle v_{i}\otimes e_{j}:i=\pm 1,j=0,-1,2,\pm 3\rangle$	21	$\langle e\rangle$
$x_{1}$	$h_{\alpha _{1}}(t)$	$\langle E,H,F,x_{5},y_{2},x_{1},x_{6},y_{6},h_{1}+2h_{2}\rangle \oplus \langle v_{i}\otimes e_{j}:i=\pm 1,j=-2,1,3\rangle$	15	$\langle e\rangle$
$x_{2}+x_{5}$	$h_{\alpha _{1}}(t^{2})h_{\alpha _{2}}(t^{4})$	$\langle E,H,F,x_{6},x_{2}+x_{5},x_{3},x_{4}\rangle \oplus \langle v_{i}\otimes e_{j}:i=\pm 1,j=0,2,3\rangle$	13	$\langle e,x_{6}\rangle$
$x_{1}+x_{2}$	$h_{\alpha _{1}}(t^{6})h_{\alpha _{2}}(t^{10})$	$\langle E,H,F,x_{6},x_{1}+x_{2}\rangle \oplus \langle v_{i}\otimes e_{3}:i=\pm 1\rangle$	7	$\langle e,x_{6}\rangle$

**Table 9:** Cocharacters and $\mathfrak {g}^{e}$ for $\mathfrak {g}=G(3)$

Representatives of nilpotent orbits $e\in \mathfrak {g}_{\bar{0}}$	Cocharacter $\tau$	$\mathfrak {g}^{e}$	$\dim \mathfrak {g}^{e}$	$\mathfrak {z}\left( \mathfrak {g}^{e}\right)$
*E*	$h_{\alpha _{0}}(t)$	$\langle E\rangle \oplus G_{2}\oplus \langle v_{1}\otimes e_{j}:j=0,\pm 1,\pm 2,\pm 3\rangle$	22	$\langle e\rangle$
$E+x_{2}$	$h_{\alpha _{0}}(t)h_{\alpha _{2}}(t)$	$\langle E\rangle \oplus \langle 2h_{1}+3h_{2},x_{2},x_{3},x_{6},y_{1},y_{5},x_{4},y_{4}\rangle \oplus \langle v_{1}\otimes e_{2},v_{1}\otimes e_{-1},v_{1}\otimes e_{\pm 3},v_{1}\otimes e_{0},v_{1}\otimes e_{-2}+v_{-1}\otimes e_{-1},v_{1}\otimes e_{1}-v_{-1}\otimes e_{2}\rangle$	16	$\langle e\rangle$
$E+x_{1}$	$h_{\alpha _{0}}(t)h_{\alpha _{1}}(t)$	$\langle E\rangle \oplus \langle x_{5},y_{2},x_{1},x_{6},y_{6},h_{1}+2h_{2}\rangle \oplus \langle v_{1}\otimes e_{1},v_{1}\otimes e_{3},v_{1}\otimes e_{-2},v_{1}\otimes e_{0}-v_{-1}\otimes e_{1},v_{1}\otimes e_{-3}-v_{-1}\otimes e_{-2},v_{1}\otimes e_{2}+v_{-1}\otimes e_{3}\rangle$	13	$\langle e\rangle$
$E+(x_{2}+x_{5})$	$h_{\alpha _{0}}(t)h_{\alpha _{1}}(t^{2})h_{\alpha _{2}}(t^{4})$	$\langle E\rangle \oplus \langle x_{6},x_{2}+x_{5},x_{3},x_{4}\rangle \oplus \langle v_{1}\otimes e_{3},v_{1}\otimes e_{2},v_{1}\otimes e_{0},v_{1}\otimes e_{1}-v_{-1}\otimes e_{2},6v_{-1}\otimes e_{3}-v_{1}\otimes e_{-1}\rangle$	10	$\langle e,x_{6}\rangle$
$E+(x_{1}+x_{2})$	$h_{\alpha _{0}}(t)h_{\alpha _{1}}(t^{6})h_{\alpha _{2}}(t^{10})$	$\langle E\rangle \oplus \langle x_{6},x_{1}+x_{2}\rangle \oplus \langle v_{1}\otimes e_{3},v_{-1}\otimes e_{3}+v_{1}\otimes e_{2}\rangle$	5	$\langle e,x_{6},v_{1}$ $\otimes e_{3}\rangle$

We know that the map $\mathfrak {g}_{\bar{0}}\times \mathfrak {g}_{\bar{0}}\rightarrow \mathfrak {g}_{\bar{0}}$ is a Lie bracket and the Lie superbracket $[\cdot ,\cdot ]:\mathfrak {g}_{\bar{0}}\times \mathfrak {g}_{\bar{1}}\rightarrow \mathfrak {g}_{\bar{1}}$ is defined by $[x+y,v_{i}\otimes e_{j}]=xv_{i}\otimes e_{j}+v_{i}\otimes ye_{j}$ for $x\in \mathfrak {sl}_{2}(\mathbb {K})$, $y\in G_{2}$ and $v_{i}\otimes e_{j}\in \mathfrak {g}_{\bar{1}}$. The Lie superbracket $[\cdot ,\cdot ]:\mathfrak {g}_{\bar{1}}\times \mathfrak {g}_{\bar{1}}\rightarrow \mathfrak {g}_{\bar{0}}$ over a field of zero characteristic is calculated explicitly in [7, Subsection 5.1]. Note that the structure remains the same when we work in the field $\mathbb {K}$ with $\textrm{char}(\mathbb {K})=p>3$ and we adopt it to calculate a basis for $\mathfrak {g}^{e}$ and $\mathfrak {z}(\mathfrak {g}^{e})$.

It is clear that representatives of nilpotent orbits in $\mathfrak {sl}_{2}(\mathbb {K})$ up to conjugation by $\textrm{SL}_{2}(\mathbb {K})$ are 0 and *E*. Based on [16, Section 11], representatives of nilpotent orbits in Lie algebra $G_{2}$ up to conjugation by Lie group $G_{2}$ are 0, $x_{1}$, $x_{2}$, $x_{2}+x_{5}$ and $x_{1}+x_{2}$. Representatives of nilpotent orbits $e\in G(3)$ are listed in Tables 8–9. In these tables, we also give the cocharacter $\tau$ associated to *e* and basis elements for $\mathfrak {g}^{e}$ and $\mathfrak {z}\left( \mathfrak {g}^{e}\right)$. Note that in the second column of Tables 8 and 9, we denote by $\alpha _{0}=2\delta$, $\alpha _{1}=\varepsilon _{1}$, $\alpha _{2}=\varepsilon _{2}-\varepsilon _{1}$ where $2\delta ,\varepsilon _{1},\varepsilon _{2}-\varepsilon _{1}$ are even roots in the root system for $\mathfrak {g}$ as given in Sect. 2.2. After Table 9, we take the case when $e=x_{2}$ as an example to show our calculation explicitly. The other cases are obtained using a similar approach.

When $e=x_{2}$, we first compute that the cocharacter associated to *e* is $\tau (t)=h_{\alpha _{2}}(t)$. According to [16, Section 5] and [7, Section 5.3], we have that $\mathfrak {g}_{\bar{0}}^{e}=\mathfrak {sl}_{2}(\mathbb {K})\oplus \langle 2h_{1}+3h_{2},x_{2},x_{3},x_{6},y_{1},y_{5},x_{4},y_{4}\rangle$.

In order to determine $\mathfrak {g}_{\bar{1}}^{e}$, we work out the $\tau$-grading on basis elements of $\mathfrak {g}_{\bar{1}}$ in Table 10.

From the above table, we know that $\mathfrak {g}_{\bar{1}}^{e}=\mathfrak {g}_{\bar{1}}^{e}(-1)\oplus \mathfrak {g}_{\bar{1}}^{e}(0)\oplus \mathfrak {g}_{\bar{1}}^{e}(1)$. Since $[e,\mathfrak {g}_{\bar{1}}^{e}(1)]\subseteq \mathfrak {g}_{\bar{1}}^{e}(3)$ and $\mathfrak {g}_{\bar{1}}^{e}(3)=0$, we get that $\mathfrak {g}_{\bar{1}}^{e}(1)=\langle v_{i}\otimes e_{2},v_{i}\otimes e_{-1}:i=\pm 1\rangle$. Similarly, we have that $\mathfrak {g}_{\bar{1}}^{e}(0)=\langle v_{i}\otimes e_{\pm 3},v_{i}\otimes e_{0}:i=\pm 1\rangle$. Then let $x=\sum a_{i,-2}v_{i}\otimes e_{-2}+\sum a_{i,1}v_{i}\otimes e_{1}\in \mathfrak {g}_{\bar{1}}^{e}(-1)$, we have that $[e,x]=-\sum a_{i,-2}v_{i}\otimes e_{-1}+a_{i,1}v_{i}\otimes e_{2}$. This equals to zero implies that $a_{i,-2}=a_{i,1}=0$, thus $\mathfrak {g}_{\bar{1}}^{e}(-1)=0$. Hence, we deduce that $\mathfrak {g}_{\bar{1}}^{e}=\langle v_{i}\otimes e_{j}:i=\pm 1,j=0,-1,2,\pm 3\rangle$ (Table 10).

Next, we look for $\mathfrak {z}(\mathfrak {g}^{e})=\mathfrak {z}_{\bar{0}}\oplus \mathfrak {z}_{\bar{1}}$. Note that $\mathfrak {z}_{\bar{0}}=\mathfrak {z}_{\bar{0}}(2)\oplus \mathfrak {z}_{\bar{0}}(1)\oplus \mathfrak {z}_{\bar{0}}(0)$. We know that there is no centre in $\mathfrak {sl}_{2}(\mathbb {K})$. Let $y=b_{1}y_{1}+b_{3}x_{3}+b_{5}y_{5}+b_{6}x_{6}\in \mathfrak {z}_{\bar{0}}(1)$, we compute $[2h_{1}+3h_{2},y]=-b_{1}y_{1}+b_{3}x_{3}-3b_{5}y_{5}+3b_{6}x_{6}$ for some $b_{i}\in \mathbb {K}$. This equals to zero if and only if $b_{1}=b_{3}=b_{5}=b_{6}=0$. Thus $\mathfrak {z}_{\bar{0}}(1)=0$. Using a similar method, we have that $\mathfrak {z}_{\bar{0}}(0)=0$ and $\mathfrak {z}_{\bar{0}}(2)=\langle e\rangle$. For an element $z\in \mathfrak {g}_{\bar{1}}^{e}$ such that $z=\sum _{i}a_{i,2}v_{i}\otimes e_{2}+\sum _{i}a_{i,-1}v_{i}\otimes e_{-1}+\sum _{i}a_{1,3}v_{i}\otimes e_{3}+\sum _{i}a_{1,-3}v_{i}\otimes e_{-3}+\sum _{i}a_{i,0}v_{i}\otimes e_{0}$, by calculating [*H*, *z*], we obtain that $\mathfrak {z}_{\bar{1}}=0$. Therefore, we deduce that $\mathfrak {z}(\mathfrak {g}^{e})=\langle e\rangle$.

**Table 10:** The $\tau$-grading on basis elements of $\mathfrak {g}_{\bar{1}}$when $e=x_{2}$

$\tau$-grading	basis elements of $\mathfrak {g}_{\bar{1}}$
1	$v_{i}\otimes e_{2},v_{i}\otimes e_{-1}$, $i=\pm 1$
0	$v_{i}\otimes e_{\pm 3},v_{i}\otimes e_{0}$, $i=\pm 1$
$-1$	$v_{i}\otimes e_{-2},v_{i}\otimes e_{1}$, $i=\pm 1$

### Centralizer of even Nilpotent Elements in *F*(4) and its Centre

Let $\mathfrak {g}=\mathfrak {g}_{\bar{0}}\oplus \mathfrak {g}_{\bar{1}}=F(4)$. By definition, $\mathfrak {g}_{\bar{0}}=\mathfrak {sl}_{2}(\mathbb {K})\oplus \mathfrak {so}_{7}(\mathbb {K})$ and $\mathfrak {g}_{\bar{1}}=V_{2}\otimes V_{8}$ where $V_{2}=\langle v_{1}=(1,0)^{t},v_{-1}=(0,1)^{t}\rangle$ is the standard two-dimensional representation for $\mathfrak {sl}_{2}(\mathbb {K})$ and $V_{8}$ is the spin representation for $\mathfrak {so}_{7}(\mathbb {K})$. Let $V_{\mathfrak {so}}$ be the standard 7-dimensional module of $\mathfrak {so}_{7}(\mathbb {K})$ such that $\mathfrak {so}_{7}(\mathbb {K})=\mathfrak {so}(V_{\mathfrak {so}})$ and let $\beta$ be the symmetric bilinear form on $V_{\mathfrak {so}}$. We adopt the notation from [7, Section 6.1] for a basis for $V_{\mathfrak {so}}$. Note that there exists a decomposition of $V_{\mathfrak {so}}$ such that $V_{\mathfrak {so}}=W\oplus \langle e_{0}\rangle \oplus W^{*}$ where *W*, $W^{*}$ is a pair of dual maximal isotropic subspaces of $V_{\mathfrak {so}}$ corresponding to $\beta$ and $W=\langle e_{1},e_{2},e_{3}\rangle$, $W^{*}=\langle e_{-1},e_{-2},e_{-3}\rangle$. The symmetric form $\beta$ on the chosen basis elements is given by $\beta (e_{0},e_{0})=2$, $\beta (e_{0},W)=\beta (e_{0},W^{*})=0$ and $\beta (e_{i},e_{-j})=\delta _{ij}$ for $i,j\ne 0$. Denote by $e_{i,j}$ the elementary transformation which sends $e_{i}$ to $e_{j}$ and the other basis vectors to 0. Then the following elements

5.1 $$\begin{aligned} R_{i,-j}=e_{i,j}-e_{-j,-i}\text { and }R_{i,0}=2e_{i,0}-e_{0,-i}\text { for }i,j\in \{\pm 1,\pm 2,\pm 3\} \end{aligned}$$

form a basis for $\mathfrak {so}_{7}(\mathbb {K})$, see [5, Lemma 6.2.1]. Next consider a 1-dimensional vector space $\langle s\rangle$ such that $e_{-i}s=0$ for $i=1,2,3$ and $e_{0}s=s$. Then a basis for $V_{8}$ can be written as $\{s$, $e_{1}s$, $e_{2}s$, $e_{3}s$, $e_{1}e_{2}s$, $e_{1}e_{3}s$, $e_{2}e_{3}s$, $e_{1}e_{2}e_{3}s\}$, see [7, Subsection 6.2].

According to [12, Section 1.6], nilpotent orbits in $\mathfrak {so}_{7}(\mathbb {K})$ are parameterized by partition $\lambda$ such that $\lambda \in \{(7),(5,1^{2}),(3^{2},1),(3,2^{2}),(3,1^{4}),(2^{2},1^{3}),(1^{7})\}$. In Table 11, we list representatives of nilpotent orbits $e=e_{1}+e_{2}\in \mathfrak {sl}_{2}(\mathbb {K})\oplus \mathfrak {so}_{7}(\mathbb {K})$ up to conjugation by $\textrm{SL}_{2}(\mathbb {K})\times \textrm{SO}_{7}(\mathbb {K})$ such that $e_{2}$ are expressed in terms of $R_{i,j}$ in Eq. 5.1. Basis elements of $\mathfrak {so}_{7}(\mathbb {C})^{e_{2}}$ are given in [7, Table 10], note that the basis of $\mathfrak {so}_{7}(\mathbb {C})^{e_{2}}$ viewed in $\mathfrak {so}_{7}(\mathbb {K})^{e_{2}}$ is also a basis for $\mathfrak {so}_{7}(\mathbb {K})^{e_{2}}$ according to [12, Section 3.2]. We also give the cocharacter $\tau$ associated to *e* and recall basis elements for $\mathfrak {g}_{\bar{0}}^{e}$ in the table below. Note that in the second column of Table 11, we denote by $\alpha _{0}=\delta$, $\alpha _{1}=\varepsilon _{1}-\varepsilon _{2}$, $\alpha _{2}=\varepsilon _{2}-\varepsilon _{3}$ and $\alpha _{3}=\varepsilon _{3}$ where $\delta$, $\varepsilon _{1}-\varepsilon _{2}$, $\varepsilon _{2}-\varepsilon _{3}$, $\varepsilon _{3}$ are even roots in the root system for $\mathfrak {g}$ as given in Sect. 2.2.

**Table 11:** Cocharacters and $\mathfrak {g}_{\bar{0}}^{e}$ for $\mathfrak {g}=F(4)$

Representatives of nilpotent orbits $e\in \mathfrak {g}_{\bar{0}}$	Cocharacter $\tau$	$\mathfrak {g}_{\bar{0}}^{e}$	$\dim \mathfrak {g}_{\bar{0}}^{e}$
$e_{(7)}=R_{1,-2}+R_{2,-3}+R_{3,0}$	$h_{\alpha _{1}}(t^{6})h_{\alpha _{2}}(t^{10})h_{\alpha _{3}}(t^{6})$	$\mathfrak {sl}_{2}(\mathbb {K})\oplus \langle e,R_{1,0}-2R_{2,3},R_{1,2}\rangle$	6
$e_{(5,1^{2})}=R_{1,-2}+R_{2,0}$	$h_{\alpha _{1}}(t^{4})h_{\alpha _{2}}(t^{6})h_{\alpha _{3}}(t^{3})$	$\mathfrak {sl}_{2}(\mathbb {K})\oplus \langle R_{3,-3},e,R_{1,-3},R_{1,3},R_{1,2}\rangle$	8
$e_{(3^{2},1)}=R_{1,-3}+R_{2,3}$	$h_{\alpha _{1}}(t^{2})h_{\alpha _{2}}(t^{4})h_{\alpha _{3}}(t^{2})$	$\mathfrak {sl}_{2}(\mathbb {K})\oplus \langle R_{1,-1}-R_{2,-2}+R_{3,-3},$ $e,R_{2,-3},R_{2,0},R_{1,0},R_{1,3},R_{1,2}\rangle$	10
$e_{(3,2^{2})}=R_{1,0}+R_{2,3}$	$h_{\alpha _{1}}(t^{2})h_{\alpha _{2}}(t^{3})h_{\alpha _{3}}(t^{2})$	$\mathfrak {sl}_{2}(\mathbb {K})\oplus \langle R_{2,-2}-R_{3,-3},R_{2,-3},$ $R_{3,-2},2R_{1,-3}+R_{2,0},-2R_{1,-2}+R_{3,0},$ $R_{1,0},R_{2,3},R_{1,3},R_{1,2}\rangle$	12
$e_{(3,1^{4})}=R_{1,0}$	$h_{\alpha _{1}}(t^{2})h_{\alpha _{2}}(t^{2})h_{\alpha _{3}}(t)$	$\mathfrak {sl}_{2}(\mathbb {K})\oplus \langle R_{2,-2},R_{3,-3},R_{2,3},R_{2,-3},$ $R_{-3,-2},R_{3,-2},e,R_{1,2},R_{1,3},R_{1,-3},R_{1,-2}\rangle$	14
$e_{(2^{2},1^{3})}=R_{1,2}$	$h_{\alpha _{1}}(t)h_{\alpha _{2}}(t^{2})h_{\alpha _{3}}(t)$	$\mathfrak {sl}_{2}(\mathbb {K})\oplus \langle R_{1,-2},R_{1,-1}-R_{2,-2},R_{3,-3},$ $R_{2,-1},R_{-3,0},R_{3,0},R_{1,3},R_{1,0},R_{2,-3},$ $R_{2,3},R_{1,-3},R_{2,0},e\rangle$	16
$e_{(1^{7})}=0$	0	$\mathfrak {sl}_{2}(\mathbb {K})\oplus \mathfrak {so}_{7}(\mathbb {K})$	24
$E+e_{(7)}$	$h_{\alpha _{0}}(t)h_{\alpha _{1}}(t^{6})h_{\alpha _{2}}(t^{10})h_{\alpha _{3}}(t^{6})$	$\langle E,e_{(7)},R_{1,0}-2R_{2,3},R_{1,2}\rangle$	4
$E+e_{(5,1^{2})}$	$h_{\alpha _{0}}(t)h_{\alpha _{1}}(t^{4})h_{\alpha _{2}}(t^{6})h_{\alpha _{3}}(t^{3})$	$\langle E,R_{3,-3},e_{(5,1^{2})},R_{1,-3},R_{1,3},R_{1,2}\rangle$	6
$E+e_{(3^{2},1)}$	$h_{\alpha _{0}}(t)h_{\alpha _{1}}(t^{2})h_{\alpha _{2}}(t^{4})h_{\alpha _{3}}(t^{2})$	$\langle E,R_{1,-1}-R_{2,-2}+R_{3,-3},e_{(3^{2},1)},$ $R_{2,-3},R_{2,0},R_{1,0},R_{1,3},R_{1,2}\rangle$	8
$E+e_{(3,2^{2})}$	$h_{\alpha _{0}}(t)h_{\alpha _{1}}(t^{2})h_{\alpha _{2}}(t^{3})h_{\alpha _{3}}(t^{2})$	$\langle E,R_{2,-2}-R_{3,-3},R_{2,-3},R_{3,-2},$ $2R_{1,-3}+R_{2,0},-2R_{1,-2}+R_{3,0},$ $R_{1,0},R_{2,3},R_{1,3},R_{1,2}\rangle$	10
$E+e_{(3,1^{4})}$	$h_{\alpha _{0}}(t)h_{\alpha _{1}}(t^{2})h_{\alpha _{2}}(t^{2})h_{\alpha _{3}}(t)$	$\langle E,R_{2,-2},R_{3,-3},R_{2,3},R_{2,-3},R_{-3,-2},$ $R_{3,-2},e_{(3,1^{4})},R_{1,2},R_{1,3},R_{1,-3},R_{1,-2}\rangle$	12
$E+e_{(2^{2},1^{3})}$	$h_{\alpha _{0}}(t)h_{\alpha _{1}}(t)h_{\alpha _{2}}(t^{2})h_{\alpha _{3}}(t)$	$\langle E,R_{1,-2},R_{1,-1}-R_{2,-2},R_{3,-3},R_{2,-1},$ $R_{-3,0},R_{3,0},R_{1,3},R_{1,0},R_{2,-3},R_{2,3},$ $R_{1,-3},R_{2,0},e_{(2^{2},1^{3})}\rangle$	14
*E*	$h_{\alpha _{0}}(t)$	$\langle E\rangle \oplus \mathfrak {so}_{7}(\mathbb {K})$	22

We then give basis elements for $\mathfrak {g}_{\bar{1}}^{e}$ and $\mathfrak {z}(\mathfrak {g}^{e})$ in Table 12.

**Table 12:** $\mathfrak {g}_{\bar{1}}^{e}$ and $\mathfrak {z}(\mathfrak {g}^{e})$ for $\mathfrak {g}=F(4)$

Representatives of nilpotent orbits $e\in \mathfrak {g}_{\bar{0}}$	$\mathfrak {g}_{\bar{1}}^{e}$	$\dim \mathfrak {g}_{\bar{1}}^{e}$	$\mathfrak {z}(\mathfrak {g}^{e})$
$e_{(7)}$	$\langle v_{i}\otimes e_{1}e_{2}e_{3}s,v_{i}\otimes e_{1}s-v_{i}\otimes e_{2}e_{3}s:i=\pm 1\rangle$	4	$\langle e,R_{1,2}\rangle$
$e_{(5,1^{2})}$	$\langle v_{i}\otimes e_{1}e_{2}e_{3}s,v_{i}\otimes e_{1}e_{2}s:i=\pm 1\rangle$	4	$\langle e,R_{1,2}\rangle$
$e_{(3^{2},1)}$	$\langle v_{i}\otimes e_{1}e_{2}s,v_{i}\otimes e_{1}e_{2}e_{3}s,v_{i}\otimes e_{1}e_{3}s,$ $v_{i}\otimes e_{2}s:i=\pm 1\rangle$	8	$\langle e,R_{1,2}\rangle$
$e_{(3,2^{2})}$	$\langle v_{i}\otimes e_{1}e_{2}e_{3}s,v_{i}\otimes e_{1}e_{2}s,v_{i}\otimes e_{1}e_{3}s,$ $v_{i}\otimes e_{1}s-v_{i}\otimes e_{2}e_{3}s:i=\pm 1\rangle$	8	$\langle e\rangle$
$e_{(3,1^{4})}$	$\langle v_{i}\otimes e_{1}e_{2}e_{3}s,v_{i}\otimes e_{1}e_{2}s,v_{i}\otimes e_{1}e_{3}s,$ $v_{i}\otimes e_{1}s:i=\pm 1\rangle$	8	$\langle e\rangle$
$e_{(2^{2},1^{3})}$	$\langle v_{i}\otimes e_{1}e_{2}e_{3}s,v_{i}\otimes e_{1}e_{2}s,v_{i}\otimes e_{1}e_{3}s,$ $v_{i}\otimes e_{1}s,v_{i}\otimes e_{2}s,v_{i}\otimes e_{2}e_{3}s:i=\pm 1\rangle$	12	$\langle e\rangle$
$e_{(1^{7})}$	$\mathfrak {g}_{\bar{1}}$	16	$\langle e\rangle =0$
$E+e_{(7)}$	$\langle v_{1}\otimes e_{1}e_{2}e_{3}s,v_{1}\otimes e_{1}e_{2}s-v_{-1}\otimes e_{1}e_{2}e_{3}s,$ $v_{1}\otimes e_{1}s-v_{1}\otimes e_{2}e_{3}s\rangle$	3	$\langle e,R_{1,2},$ $v_{1}\otimes e_{1}e_{2}e_{3}s\rangle$
$E+e_{(5,1^{2})}$	$\langle v_{1}\otimes e_{1}e_{2}e_{3}s,v_{1}\otimes e_{1}e_{2}s,v_{1}\otimes e_{1}s$ $-v_{-1}\otimes e_{1}e_{2}s,v_{1}\otimes e_{1}e_{3}s+v_{-1}\otimes e_{1}e_{2}e_{3}s\rangle$	4	$\langle e,R_{1,2}\rangle$
$E+e_{(3^{2},1)}$	$\langle v_{1}\otimes e_{1}e_{2}s,v_{1}\otimes e_{1}e_{2}e_{3}s,v_{1}\otimes e_{2}s,$ $v_{1}\otimes e_{1}s-v_{-1}\otimes e_{1}e_{2}e_{3}s,v_{1}\otimes e_{1}e_{3}s,$ $v_{-1}\otimes e_{1}e_{2}s+v_{1}\otimes e_{2}e_{3}s\rangle$	6	$\langle e,R_{1,2}\rangle$
$E+e_{(3,2^{2})}$	$\langle v_{1}\otimes e_{1}e_{2}e_{3}s,v_{1}\otimes e_{1}e_{2}s,v_{1}\otimes e_{1}e_{3}s,$ $v_{1}\otimes e_{1}s-v_{-1}\otimes e_{1}e_{2}e_{3}s,v_{1}\otimes e_{2}e_{3}s$ $-v_{-1}\otimes e_{1}e_{2}e_{3}s,v_{1}\otimes e_{3}s+v_{-1}\otimes e_{1}e_{3}s,$ $v_{1}\otimes e_{2}s+v_{-1}\otimes e_{1}e_{2}s\rangle$	7	$\langle e\rangle$
$E+e_{(3,1^{4})}$	$\langle v_{1}\otimes e_{1}s,v_{1}\otimes e_{1}e_{3}s,v_{1}\otimes e_{1}e_{2}e_{3}s,$ $v_{1}\otimes e_{1}e_{2}s,v_{1}\otimes s-v_{-1}\otimes e_{1}s,v_{1}\otimes e_{2}s$ $+v_{-1}\otimes e_{1}e_{2}s,v_{1}\otimes e_{3}s+v_{-1}\otimes e_{1}e_{3}s,$ $v_{1}\otimes e_{2}e_{3}s-v_{-1}\otimes e_{1}e_{2}e_{3}s,\rangle$	8	$\langle e\rangle$
$E+e_{(2^{2},1^{3})}$	$\langle v_{1}\otimes e_{1}e_{2}e_{3}s,v_{1}\otimes e_{1}e_{2}s,v_{1}\otimes e_{1}s,$ $v_{1}\otimes e_{2}s,v_{1}\otimes e_{1}e_{3}s,v_{1}\otimes e_{2}e_{3}s,v_{1}\otimes s$ $-v_{-1}\otimes e_{1}e_{2}s,v_{1}\otimes e_{3}s-v_{-1}\otimes e_{1}e_{2}e_{3}s\rangle$	8	$\langle e\rangle$
*E*	$\langle v_{1}\otimes s,v_{1}\otimes e_{1}s,v_{1}\otimes e_{2}s,v_{1}\otimes e_{3}s,v_{1}\otimes e_{1}e_{2}s,$ $v_{1}\otimes e_{1}e_{3}s,v_{1}\otimes e_{2}e_{3}s,v_{1}\otimes e_{1}e_{2}e_{3}s\rangle$	8	$\langle e\rangle$

**Table 13:** The $\tau$-grading on basis elements of $\mathfrak {g}_{\bar{1}}$ when $e=e_{(5,1^{2})}$

$\tau$-grading	basis elements of $\mathfrak {g}_{\bar{1}}$
3	$v_{i}\otimes e_{1}e_{2}e_{3}s,v_{i}\otimes e_{1}e_{2}s\text { for }i=\pm 1$
1	$v_{i}\otimes e_{1}s,v_{i}\otimes e_{1}e_{3}s\text { for }i=\pm 1$
$-1$	$v_{i}\otimes e_{2}s,v_{i}\otimes e_{2}e_{3}s\text { for }i=\pm 1$
$-3$	$v_{i}\otimes s,v_{i}\otimes e_{3}s\text { for }i=\pm 1$

In the remaining part of this subsection, we give explicit calculations on finding $\mathfrak {g}^{e}$ and $\mathfrak {z}(\mathfrak {g}^{e})$ for the case $e=e_{(5,1^{2})}$. All the other cases are obtained using a similar approach (Table 13).

Since $e=e_{(5,1^{2})}=R_{1,-2}+R_{2,0}\in \mathfrak {so}_{7}(\mathbb {K})$, by calculating $[e,x]=0$ for any $x\in \mathfrak {so}_{7}(\mathbb {K})$, we obtain that $\mathfrak {so}_{7}(\mathbb {K})^{e}=\langle R_{3,-3},e,R_{1,-3},R_{1,3},R_{1,2}\rangle$ and thus $\mathfrak {g}_{\bar{0}}^{e}=\langle E,H,F\rangle \oplus \mathfrak {so}_{7}(\mathbb {K})^{e}$.

To compute $\mathfrak {g}_{\bar{1}}^{e}$, we first work out the $\tau$-grading on basis elements of $\mathfrak {g}_{\bar{1}}$ in the table below.

According to the table above, we have that $\mathfrak {g}_{\bar{1}}^{e}=\mathfrak {g}_{\bar{1}}^{e}(3)\oplus \mathfrak {g}_{\bar{1}}^{e}(1)\oplus \mathfrak {g}_{\bar{1}}^{e}(-1)\oplus \mathfrak {g}_{\bar{1}}^{e}(-3)$. We get $\mathfrak {g}_{\bar{1}}^{e}(3)=\langle v_{i}\otimes e_{1}e_{2}e_{3}s,v_{i}\otimes e_{1}e_{2}s:i=\pm 1\rangle$ directly from the grading. For any element $x=\sum _{i}a_{i,1}v_{i}\otimes e_{1}s+\sum _{i}a_{i,13}v_{i}\otimes e_{1}e_{3}s\in \mathfrak {g}_{\bar{1}}^{e}(1)$, we have that $[e,x]=\sum _{i}a_{i,1}v_{i}\otimes e_{1}e_{2}s-\sum _{i}a_{i,13}v_{i}\otimes e_{1}e_{2}e_{3}s$. Thus, $[e,x]=0$ if and only if $a_{i,1}=a_{i,13}=0$. Hence, we know that $\mathfrak {g}_{\bar{1}}^{e}(1)=0$. Applying a similar calculation, we obtain that $\mathfrak {g}_{\bar{1}}^{e}(-1)=\mathfrak {g}_{\bar{1}}^{e}(-3)=0$ and therefore $\mathfrak {g}_{\bar{1}}^{e}=\mathfrak {g}_{\bar{1}}^{e}(3)=\langle v_{i}\otimes e_{1}e_{2}e_{3}s,v_{i}\otimes e_{1}e_{2}s:i=\pm 1\rangle$ (Table 14).

Next, we determine $\mathfrak {z}(\mathfrak {g}^{e})=\mathfrak {z}_{\bar{0}}\oplus \mathfrak {z}_{\bar{1}}$. Clearly, $e\in \mathfrak {z}_{\bar{0}}$. Let $y=b_{1}R_{3,-3}+b_{2}e+b_{3}R_{1,-3}+b_{4}R_{1,3}+b_{5}R_{1,2}$ be an element in $\mathfrak {z}_{\bar{0}}$. Then $[R_{3,-3},y]=-b_{3}R_{1,-3}+b_{4}R_{1,3}$, this equals to zero implies that $b_{3}=b_{4}=0$. Similarly by computing $[R_{1,3},y]$, we deduce that $b_{1}=0$. Note that $R_{1,2}\in \mathfrak {g}^{e}(6)$ and $\mathfrak {g}^{e}(6+j)=0$ for all *j* such that $\mathfrak {g}^{e}(j)\ne 0$. Thus, we deduce that $R_{1,2}\in \mathfrak {z}_{\bar{0}}$ and $\mathfrak {z}_{\bar{0}}=\langle e,R_{e_{1},e_{2}}\rangle$. To determine $\mathfrak {z}_{\bar{1}}$, let $z=\sum _{i}a_{i,123}v_{i}\otimes e_{1}e_{2}e_{3}+\sum _{i}a_{i,12}v_{i}\otimes e_{1}e_{2}s\in \mathfrak {z}_{\bar{1}}$. Then, $[H,z]=\sum _{i}ia_{i,123}v_{i}\otimes e_{1}e_{2}e_{3}+\sum _{i}ia_{i,12}v_{i}\otimes e_{1}e_{2}s$, this equals to zero if and only if $a_{i,123}=a_{i,12}=0$. Hence we deduce that $\mathfrak {z}_{\bar{1}}=0$. Therefore, we conclude that $\mathfrak {z}(\mathfrak {g}^{e})=\langle e,R_{e_{1},e_{2}}\rangle$ (Table 15).

## Reachability and the Panyushev Property for Exceptional Lie Superalgebras

Let $\mathfrak {g}=\mathfrak {g}_{\bar{0}}\oplus \mathfrak {g}_{\bar{1}}$ be one of exceptional Lie superalgebras $D(2,1;\alpha )$, *G*(3) and *F*(4) over $\mathbb {K}$. With the structure of $\mathfrak {g}^{e}$ obtained in Sect. 5, we further investigate some properties of nilpotent elements $e\in \mathfrak {g}_{\bar{0}}$. Recall as in Introduction that *e* is called *reachable* if $e\in [\mathfrak {g}^{e},\mathfrak {g}^{e}]$ and is called *strongly reachable* if $\mathfrak {g}^{e}=[\mathfrak {g}^{e},\mathfrak {g}^{e}]$. The element *e* is said to satisfy the *Panyushev property* if in the $\tau$-grading $\mathfrak {g}^{e}=\bigoplus _{j\ge 0}\mathfrak {g}(j)$, the subalgebra $\mathfrak {g}^{e}(\ge 1)=\bigoplus _{j\ge 1}\mathfrak {g}(j)$ is generated by $\mathfrak {g}^{e}(1)$.

In this section, we adopt notation given in Sect. 5 for representatives of nilpotent orbits $e\in \mathfrak {g}_{\bar{0}}$ and basis elements of $\mathfrak {g}^{e}$. For each $e\in \mathfrak {g}_{\bar{0}}$, we identify whether it is reachable, strongly reachable or satisfies the Panyushev property in the following table. Note that the classification is the same as the case of zero characteristic except for $\mathfrak {g}=G(3)$, $e=E+x_{1}$, in which case, we have that *e* is reachable whenever $p>5$.

**Table 14:** Reachable, strongly reachable and Panyushev elements in $D(2,1;\alpha )$

Lie superalgebra $\mathfrak {g}$	Representatives of nilpotent orbits $e\in \mathfrak {g}_{\bar{0}}$	Reachable	Strongly reachable	Satisfying the Panyushev property
$D(2,1;\alpha )$	0	Yes	Yes	Yes
	$E_{1}$, $E_{2}$, $E_{3}$	Yes	Yes	Yes
	$E_{1}+E_{2}$, $E_{2}+E_{3}$, $E_{1}+E_{3}$	No	No	No
	$E_{1}+E_{2}+E_{3}$	Yes	No	Yes

**Table 15:** Reachable, strongly reachable and Panyushev elements in *G*(3)

Lie superalgebra $\mathfrak {g}$	Representatives of nilpotent orbits $e\in \mathfrak {g}_{\bar{0}}$	Reachable	Strongly reachable	Satisfying the Panyushev property
*G*(3)	0	Yes	Yes	Yes
	$x_{2}$	Yes	Yes	Yes
	$x_{1}$	Yes	Yes	No
	$x_{2}+x_{5}$	No	No	No
	$x_{1}+x_{2}$	No	No	No
	*E*	Yes	Yes	Yes
	$E+x_{2}$	Yes	Yes	Yes
	$E+x_{1}$	Yes except $p=5$	No	No
	$E+(x_{2}+x_{5})$	Yes	No	Yes
	$E+(x_{1}+x_{2})$	No	No	No

In the remaining part of this section, we explain our calculations explicitly for the following three cases: (1) $\mathfrak {g}=D(2,1;\alpha )$, $e=E_{1}+E_{2}+E_{3}$; (2) $\mathfrak {g}=G(3)$, $e=E+x_{1}$; and (3) $\mathfrak {g}=F(4)$, $e=E+e_{(3^{2},1)}$. Note that all other cases can be done using a similar approach.

- $$\mathfrak {g}=D(2,1;\alpha )$$, $$e=E_{1}+E_{2}+E_{3}$$

Based on Table 6, we have that $\mathfrak {g}^{e}=\mathfrak {g}^{e}(1)\oplus \mathfrak {g}^{e}(2)\oplus \mathfrak {g}^{e}(3)$ where $\mathfrak {g}^{e}(1)=\langle v_{1,1,-1}-v_{-1,1,1},v_{1,-1,1}-v_{-1,1,1}\rangle$, $\mathfrak {g}^{e}(2)$=$\langle E_{1},E_{2},E_{3}\rangle$ and $\mathfrak {g}^{e}(3)=\langle v_{1,1,1}\rangle$.

**Table 16:** Reachable, strongly reachable and Panyushev elements in *F*(4)

Lie superalgebra $\mathfrak {g}$	Representatives of nilpotent orbits $e\in \mathfrak {g}_{\bar{0}}$	Reachable	Strongly reachable	Satisfying the Panyushev property
*F*(4)	$e_{(7)}$	No	No	No
	$e_{(5,1^{2})}$	No	No	No
	$e_{(3^{2},1)}$	No	No	No
	$e_{(3,2^{2})}$	Yes	Yes	Yes
	$e_{(3,1^{4})}$	Yes	Yes	Yes
	$e_{(2^{2},1^{3})}$	Yes	Yes	Yes
	$e_{(1^{7})}$	Yes	Yes	Yes
	$E+e_{(7)}$	No	No	No
	$E+e_{(5,1^{2})}$	No	No	No
	$E+e_{(3^{2},1)}$	Yes	No	Yes
	$E+e_{(3.2^{2})}$	Yes	No	Yes
	$E+e_{(3.1^{4})}$	No	No	No
	$E+e_{(2^{2}.1^{3})}$	Yes	Yes	Yes
	*E*	Yes	Yes	Yes

**Table 17:** $\tau$-grading on $\mathfrak {g}^{e}$ when $e=E+x_{1}$

$\mathfrak {g}^{e}(j)$	Basis elements of $\mathfrak {g}^{e}(j)$
$\mathfrak {g}^{e}(0)$	$x_{6},y_{6},h_{1}+2h_{2},v_{1}\otimes e_{-3}-v_{-1}\otimes e_{-2},v_{1}\otimes e_{2}+v_{-1}\otimes e_{3}$
$\mathfrak {g}^{e}(1)$	$v_{1}\otimes e_{0}-v_{-1}\otimes e_{1}$
$\mathfrak {g}^{e}(2)$	$E,x_{1},v_{1}\otimes e_{3},v_{1}\otimes e_{-2}$
$\mathfrak {g}^{e}(3)$	$x_{5},y_{2},v_{1}\otimes e_{1}$

We first check whether $\mathfrak {g}^{e}(\ge 1)$ is generated by $\mathfrak {g}^{e}(1)$. Recall that the Lie superbracket $[\cdot ,\cdot ]:\mathfrak {g}_{\bar{1}}\times \mathfrak {g}_{\bar{1}}\rightarrow \mathfrak {g}_{\bar{0}}$ depends on $\sigma _{1}$, $\sigma _{2}$, $\sigma _{3}$ such that $\sigma _{1}+\sigma _{2}+\sigma _{3}=0$ and $\sigma _{i}\ne 0$ for $i=1,2,3$. We compute that $[v_{1,1,-1}-v_{-1,1,1},v_{1,1,-1}-v_{-1,1,1}]=4\sigma _{2}E_{2}$, $[v_{1,-1,1}-v_{-1,1,1},v_{1,-1,1}-v_{-1,1,1}]=4\sigma _{3}E_{3}$, $[v_{1,1,-1}-v_{-1,1,1},v_{1,-1,1}-v_{-1,1,1}]=-2\sigma _{1}E_{1}+2\sigma _{2}E_{2}+2\sigma _{3}E_{3}$ and $[E_{2},v_{1,-1,1}-v_{-1,1,1}]=v_{1,1,1}$. Hence, we have that $\mathfrak {g}^{e}(\ge 1)$ is generated by $\mathfrak {g}^{e}(1)$.

We have that $e=-\frac{1}{2\sigma _{1}}[v_{1,1,-1}-v_{-1,1,1},v_{1,-1,1}-v_{-1,1,1}]+\frac{\sigma _{1}+\sigma _{2}}{4\sigma _{1}\sigma _{2}}[v_{1,1,-1}-v_{-1,1,1},v_{1,1,-1}-v_{-1,1,1}]+\frac{\sigma _{1}+\sigma _{3}}{4\sigma _{1}\sigma _{3}}[v_{1,-1,1}-v_{-1,1,1},v_{1,-1,1}-v_{-1,1,1}]$. Clearly $-\frac{1}{2\sigma _{1}}\ne 0$ and since $\sigma _{i}\ne 0$ for $i=1,2,3$, we have that $\frac{\sigma _{1}+\sigma _{2}}{4\sigma _{1}\sigma _{2}}=\frac{-\sigma _{3}}{4\sigma _{1}\sigma _{2}}\ne 0$. Similarly $\frac{\sigma _{1}+\sigma _{3}}{4\sigma _{1}\sigma _{3}}\ne 0$. Therefore, we deduce that $e\in [\mathfrak {g}^{e},\mathfrak {g}^{e}]$. However, we have that *e* is not strongly reachable as $\mathfrak {g}^{e}(1)\not \subseteq [\mathfrak {g}^{e},\mathfrak {g}^{e}]$.

- $$\mathfrak {g}=G(3)$$, $$e=E+x_{1}$$

According to Table 9, we know that $\mathfrak {g}^{e}=\mathfrak {g}^{e}(0)\oplus \mathfrak {g}^{e}(1)\oplus \mathfrak {g}^{e}(2)\oplus \mathfrak {g}^{e}(3)$ where basis elements of $\mathfrak {g}^{e}(j)$ for $j=0,1,2,3$ are given in the following table.

Since $\dim \mathfrak {g}^{e}(1)=1$, in order to check whether *e* satisfies the Panyushev property, we only need to calculate $[v_{1}\otimes e_{0}-v_{-1}\otimes e_{1},v_{1}\otimes e_{0}-v_{-1}\otimes e_{1}]=-8E+8x_{1}$. This implies that $\mathfrak {g}^{e}(\ge 1)$ is not generated by $\mathfrak {g}^{e}(1)$ as we cannot obtain all basis elements of $\mathfrak {g}^{e}(2)$ from commutators between basis elements of $\mathfrak {g}^{e}(1)$. Hence, the Panyushev property does not hold for this case (Table 16).

Note that $e\in [\mathfrak {g}^{e},\mathfrak {g}^{e}]$ if and only if $e\in [\mathfrak {g}_{\bar{1}}^{e}(2),\mathfrak {g}_{\bar{1}}^{e}(0)]+[\mathfrak {g}_{\bar{1}}^{e}(1),\mathfrak {g}_{\bar{1}}^{e}(1)]$. We have calculated that $[\mathfrak {g}_{\bar{1}}^{e}(1),\mathfrak {g}_{\bar{1}}^{e}(1)]=\langle -8E+8x_{1}\rangle$. Then, we calculate that $[v_{1}\otimes e_{3},v_{1}\otimes e_{-3}-v_{-1}\otimes e_{-2}]=[v_{1}\otimes e_{-2},v_{1}\otimes e_{2}+v_{-1}\otimes e_{3}]=16E+4x_{1}$. This implies that $[\mathfrak {g}_{\bar{1}}^{e}(2),\mathfrak {g}_{\bar{1}}^{e}(0)]=\langle 16E+4x_{1}\rangle$. Thus, we have that

$$ e=\frac{3}{40}[v_{1}\otimes e_{0}-v_{-1}\otimes e_{1},v_{1}\otimes e_{0}-v_{-1}\otimes e_{1}]+\frac{1}{10}[v_{1}\otimes e_{3},v_{1}\otimes e_{-3}-v_{-1}\otimes e_{-2}]. $$

The above equality holds if and only if $\textrm{char}(\mathbb {K})=p\ne 5$. Hence, when $p\ne 5$, we have that $e\in [\mathfrak {g}^{e},\mathfrak {g}^{e}]$, i.e. *e* is reachable. When $p=5$, we have that $e\notin [\mathfrak {g}^{e}(2),\mathfrak {g}^{e}(0)]+[\mathfrak {g}^{e}(1),\mathfrak {g}^{e}(1)]$ and thus *e* is not reachable (Table 17).

**Table 18:** $\tau$-grading on $\mathfrak {g}^{e}$ when $e=E+e_{(3^{2},1)}$

$\mathfrak {g}^{e}(j)$	Basis elements of $\mathfrak {g}^{e}(j)$
$\mathfrak {g}^{e}(0)$	$R_{1,-1}-R_{2,-2}+R_{3,-3}$
$\mathfrak {g}^{e}(1)$	$v_{1}\otimes e_{1}s-v_{-1}\otimes e_{1}e_{2}e_{3}s,v_{1}\otimes e_{2}s,v_{-1}\otimes e_{1}e_{2}s+v_{1}\otimes e_{2}e_{3}s,v_{1}\otimes e_{1}e_{3}s$
$\mathfrak {g}^{e}(2)$	$E,e_{(3^{2},1)},R_{2,-3},R_{2,0},R_{1,0},R_{1,3}$
$\mathfrak {g}^{e}(3)$	$v_{1}\otimes e_{1}e_{2}s,v_{1}\otimes e_{1}e_{2}e_{3}s$
$\mathfrak {g}^{e}(4)$	$R_{1,2}$

However, we have that *e* is not strongly reachable because we cannot obtain the basis element of $\mathfrak {g}^{e}(1)$ from $[\mathfrak {g}^{e},\mathfrak {g}^{e}]$.

- $$\mathfrak {g}=F(4)$$, $$e=E+e_{(3^{2},1)}$$

Based on Tables 11 and 12, we know that $\mathfrak {g}^{e}=\mathfrak {g}^{e}(0)\oplus \mathfrak {g}^{e}(1)\oplus \mathfrak {g}^{e}(2)\oplus \mathfrak {g}^{e}(3)\oplus \mathfrak {g}^{e}(4)$ where basis elements of $\mathfrak {g}^{e}(j)$ for $j=0,1,2,3,4$ are given in Table 18.

Let $x=v_{1}\otimes e_{1}s-v_{-1}\otimes e_{1}e_{2}e_{3}s$ and $y=v_{-1}\otimes e_{1}e_{2}s+v_{1}\otimes e_{2}e_{3}s$. We compute $[x,x]=R_{1,0}$, $[x,v_{1}\otimes e_{2}s]=\frac{1}{2}R_{2,0}$, $[x,v_{1}\otimes e_{1}e_{3}s]=R_{1,3}$, $[x,y]=e_{(3^{2},1)}-6E$, $[v_{1}\otimes e_{2}s,v_{1}\otimes e_{1}e_{3}s]=6E$ and $[v_{1}\otimes e_{2}s,y]=R_{2,-3}$. Thus, we have that $[\mathfrak {g}^{e}(1),\mathfrak {g}^{e}(1)]=\mathfrak {g}^{e}(2)$. We further calculate that $[R_{1,0},v_{1}\otimes e_{2}s]=-v_{1}\otimes e_{1}e_{2}s$ and $[R_{1,0},y]=v_{1}\otimes e_{1}e_{2}e_{3}s$. Hence, we have that $[\mathfrak {g}^{e}(2),\mathfrak {g}^{e}(1)]=\mathfrak {g}^{e}(3)$. Similarly $[\mathfrak {g}^{e}(3),\mathfrak {g}^{e}(1)]=\mathfrak {g}^{e}(4)$ as $[v_{1}\otimes e_{1}e_{2}e_{3}s,y]=R_{1,2}$. Therefore, we conclude that $\mathfrak {g}^{e}(\ge 1)$ is generated by $\mathfrak {g}^{e}(1)$.

Note that $e=[x,y]+\frac{7}{6}[v_{1}\otimes e_{2}s,v_{1}\otimes e_{1}e_{3}s]$. Hence, we have that *e* is reachable (Table 18).

Since $\mathfrak {g}^{e}(0)$ is 1-dimensional, we have that the grading 0 subspace of $[\mathfrak {g}^{e},\mathfrak {g}^{e}]$ equals to 0. Hence, we cannot obtain the basis element of $\mathfrak {g}^{e}(0)$ from $[\mathfrak {g}^{e},\mathfrak {g}^{e}]$ and thus *e* is not strongly reachable.
